# Circadian-Related Heteromerization of Adrenergic and Dopamine D_4_ Receptors Modulates Melatonin Synthesis and Release in the Pineal Gland

**DOI:** 10.1371/journal.pbio.1001347

**Published:** 2012-06-19

**Authors:** Sergio González, David Moreno-Delgado, Estefanía Moreno, Kamil Pérez-Capote, Rafael Franco, Josefa Mallol, Antoni Cortés, Vicent Casadó, Carme Lluís, Jordi Ortiz, Sergi Ferré, Enric Canela, Peter J. McCormick

**Affiliations:** 1 Centro de Investigación Biomédica en Red sobre Enfermedades Neurodegenerativas (CIBERNED), University of Barcelona, Barcelona, Spain; 2 Department of Biochemistry and Molecular Biology, Faculty of Biology, University of Barcelona, Barcelona, Spain; 3 Neuroscience Institute and Department of Biochemistry and Molecular Biology, Faculty of Medicine, Universitat Autònoma de Barcelona, Bellaterra, Spain; 4 National Institute on Drug Abuse, Intramural Research Program, National Institutes of Health, Department of Health and Human Services, Baltimore, Maryland, United States of America; University of Geneva, Switzerland

## Abstract

The role of the pineal gland is to translate the rhythmic cycles of night and day encoded by the retina into hormonal signals that are transmitted to the rest of the neuronal system in the form of serotonin and melatonin synthesis and release. Here we describe that the production of both melatonin and serotonin by the pineal gland is regulated by a circadian-related heteromerization of adrenergic and dopamine D_4_ receptors. Through α_1__B_-D_4_ and β_1_-D_4_ receptor heteromers dopamine inhibits adrenergic receptor signaling and blocks the synthesis of melatonin induced by adrenergic receptor ligands. This inhibition was not observed at hours of the day when D_4_ was not expressed. These data provide a new perspective on dopamine function and constitute the first example of a circadian-controlled receptor heteromer. The unanticipated heteromerization between adrenergic and dopamine D_4_ receptors provides a feedback mechanism for the neuronal hormone system in the form of dopamine to control circadian inputs.

## Abstract 2

Dopamine and adrenergic receptor complexes form under a circadian-regulated cycle and directly modulate melatonin synthesis and release from the pineal gland.

## Author Summary

Animals respond to cycles of light and dark with patterns in sleeping, feeding, body temperature alterations, and other biological functions. The pineal gland translates these light signals received from the retina into a language understandable to the rest of the body through the rhythmic synthesis and release of melatonin in response to the light and dark cycle. This process is controlled by adrenergic receptors. One impressive and mysterious aspect of the system is the rapid ability of rhythmic melatonin production and/or degradation to respond to changes in the cycle. In this study, we demonstrate that part of this response is due to the formation of receptor-receptor complexes (heteromers) between the adrenergic receptors α_1B_ or β_1_ and the D_4_ dopamine receptor. Using both biochemical and biophysical methods in transfected cells and in ex vivo tissue we show that dopamine, a neurotransmitter, inhibits adrenergic receptor signaling through these heteromers. This inhibition causes a dramatic decrease in melatonin production of the pineal gland. We postulate that these heteromers provide a rapid feedback mechanism for the neuronal hormone system to modulate circadian-controlled outputs.

## Introduction

Dopamine receptors are G protein-coupled receptors (GPCRs) that consist of two major families, the D_1_-like and D_2_-like receptors. D_1_-like receptors include D_1_ and D_5_ subtypes that are known to stimulate adenylate cyclase activity via a G_s_ mechanism and D_2_-like receptors include D_2_, D_3_, and D_4_ subtypes that inhibit adenylate cyclase activity via a G_i_ mechanism [1]. Of these subtypes, D_1_ and D_2_ and their heteromers constitute the most abundant in the brain [2]–[4]. The function of the other dopamine receptor subtypes has been more difficult to determine. The dopamine D_4_ receptor was discovered 20 years ago and initially drew a lot of attention in view of its significantly higher affinity for the atypical antipsychotic clozapine compared to the previously discovered D_2_ and D_3_ receptors [5],[6]. In the retina, D_4_ receptors modulate phototransduction through a mechanism that requires cAMP [7]. It has been described that *Drd4* is the dominant dopamine receptor gene expressed in the rat pineal gland and that it is expressed in pinealocytes and retina at levels that are greater than in other tissues [8]. Rat pineal *Drd4 mRNA* expression was found to be circadian in nature and under photoneural control [8],[9]. In the pineal gland, mRNA expression for D_4_ receptors has been shown to be tightly regulated and stimulated by norepinephrine through a mechanism involving thyroid hormone [8]. Nevertheless, the amount of D_4_ receptor protein expression or function in the pineal gland is currently not known. In this study, the primary issue under consideration is whether or not dopamine D_4_ receptor is active within the pineal gland and what is the physiological role of agonist binding to D_4_ receptors with respect to pineal gland function.

The role of the pineal gland is to translate light inputs from the retina into chemical signals for the rest of the body. This is achieved via production and secretion of melatonin by the pineal gland. Melatonin production occurs on a night/day cycle and is heavily dependent on the concentration of serotonin (5-HT) [10]–[14]. The β_1_ and α_1B_ adrenergic receptors are the main receptors that control melatonin production by different mechanisms. One of them is to control the availability of 5-HT, the melatonin precursor, by increasing both the activity of tryptophan hydroxylase (TPH) and the release of 5-HT. Another is via a strict regulation of the enzyme that converts 5-HT to melatonin, the arylalkylamine N-acetyltransferase (AANAT) [15]–[18]. Despite tight regulation by the adrenergic receptors it is unclear what limits the nighttime and daytime rates of melatonin and 5-HT production. We hypothesized that one important role of dopamine D_4_ receptors in the pineal gland can be the modulation of β_1_ and α_1B_ adrenergic receptor function. One possibility for such a modulation could be through a concept becoming widely accepted for GPCRs, the modulation of function through receptor heteromer formation [19]–[29]. A receptor heteromer is a macromolecular complex composed of at least two functional receptor units with biochemical properties that are demonstrably different from those of its individual receptors [30]. Here, using a combination of approaches including biophysical, molecular and cellular biology, and metabolic assays from cultured cells to whole, intact, pineal gland, we explored the possibility that D_4_ receptor might modify adrenergic receptor function through direct receptor-receptor interaction. We report, to our knowledge, the first heteromer between dopamine and adrenergic receptors, provide new data that adrenergic receptor control of 5-HT levels can be modulated via the D_4_ receptor and show that D_4_-adrenergic receptor regulation can alter melatonin production from the pineal gland.

## Results

### D_4_ Receptors Are Functional in the Pineal Gland

**Figure 1:**
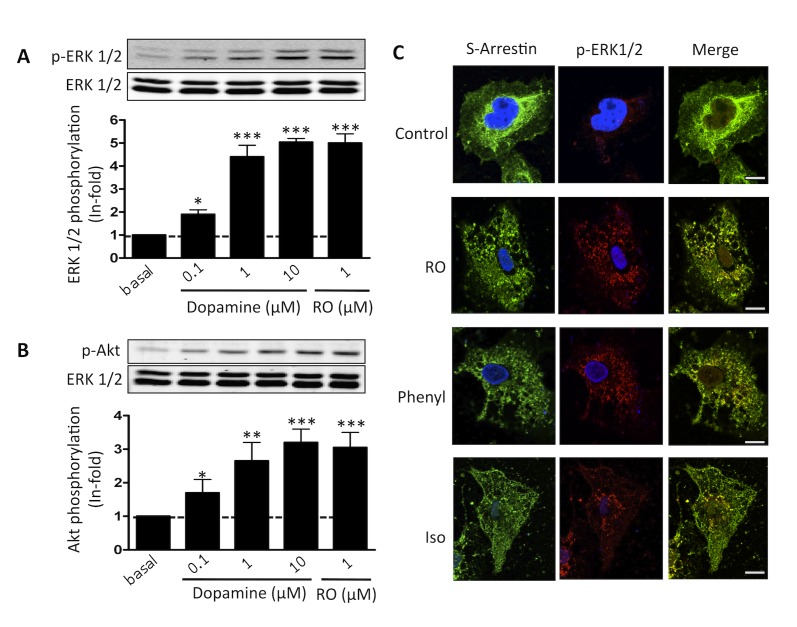
Functionality of dopamine D_4_ receptors in pineal gland and pinealocytes. Pineal glands extracted at 9:00 h were treated for 10 min with increasing amounts of dopamine or with 1 µM of RO 10-5824 (RO). The immunoreactive bands, corresponding to ERK 1/2 (Thr^183^-Tyr^185^) phosphorylation (A) and Akt (Ser^473^) phosphorylation (B), of two separate experiments performed in duplicate were quantified and values represent the mean ± S.D. of the fold increase relative to basal levels found in untreated cells. Significant differences with respect to basal levels were determined by one-way ANOVA followed by a Dunnett's multiple comparison post hoc test (*p<0.05, **p<0.01, and ***p<0.001). A representative Western blot is shown at the top (see Materials and Methods). (C) Pinealocytes were isolated from pineal glands extracted at 9:00 h and were treated with medium (Control), 1 µM of RO 10-5824 (RO), 1 µM phenylephrine (Phenyl), or 1 µM isoproterenol (Iso) for 10 min before labeling with anti-S-arrestin (green) and anti-phospho-ERK1/2 (red), as indicated in Materials and Methods. Cell nuclei were stained with DAPI (blue). Scale bar, 5 µm.

The expression of D_4_ receptor mRNA in the pineal gland during the dark period has been described, but the functional role of the protein is unknown [8],[31]. Thus we first assessed whether the receptor was active in the pineal gland. Pineal glands dissected from rats 1 h from the start of the light period were stimulated with increasing concentrations of dopamine or with the D_4_ receptor agonist RO 10-5824 and the levels of p-ERK 1/2 and p-Akt/PKB were determined. Dopamine increased both p-ERK 1/2 and p-Akt/PKB to a similar extent as RO 10-5824 (Figure 1A and B). Moreover, primary cultures of pinealocytes stimulated with RO 10-5824, the adrenergic α_1_ receptor agonist phenylephrine, or the adrenergic β receptor agonist isoproterenol, showed signaling via p-ERK 1/2 (Figure 1C, red staining). The subcellular distribution of the pinealocyte marker S-arrestin (green staining) in the absence of ligands was diffuse, suggesting cytosolic localization, and in the presence of ligands was found in punctate structures, indicating recruitment to membrane structures. In addition, these punctate structures co-localized with the p-ERK 1/2, confirming receptor activation, since endosomes containing receptor-arrestin complexes are known to serve as a signaling platform for p-ERK 1/2 (Figure 1C) [32]. Thus, in both intact pineal gland and isolated pinealocytes, D_4_ receptors are functional.

### D_4_ Receptors Form Heteromers with α_1B_ and β_1_ Receptors in Transfected Cells

**Figure 2:**
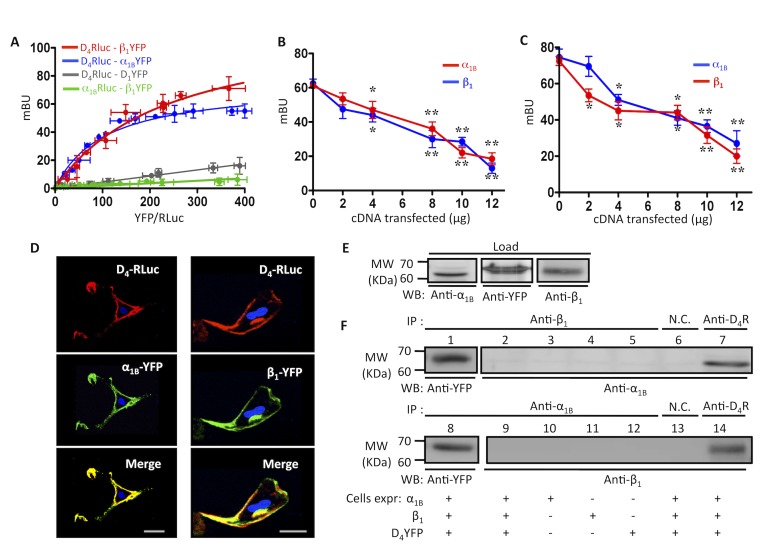
D_4_ receptors form heteromers with α_1B_ and β_1_ receptors in transfected cells. (A) BRET saturation curves were performed in HEK-293T cells co-expressing a constant amount of D_4_-RLuc construct (2 µg of plasmid transfected) and increasing amounts of β_1_-YFP construct (0.4–5 µg plasmid transfected, red), α_1B_-YFP construct (0.4–5 µg of plasmid transfected, blue), or D_1_-YFP construct (1–4 µg of plasmid transfected, gray) or with cells co-expressing a constant amount of α_1B_-RLuc construct (3 µg of plasmid transfected) and increasing amounts of β_1_-YFP construct (0.4–5 µg of plasmid transfected, green). Both fluorescence and luminescence of each sample were measured prior to every experiment to confirm equal expression of Rluc construct (∼100,000 luminescence units) while monitoring the increase of YFP construct expression (2,000 to 40,000 fluorescence units). Milli BRET Units (mBU) are BRET ratio (see Materials and Methods)×1,000 and are expressed as means ± S.D. of five different experiments grouped as a function of the amount of BRET acceptor normalized with respect to the BRET donor (YFP/RLuc). (B and C) BRET was determined in HEK-293T cells expressing a constant amount of D_4_-RLuc construct (2 µg of plasmid transfected) and (B) α_1B_-YFP construct (4 µg of plasmid transfected) or (C) β_1_-YFP construct (4 µg of plasmid transfected) and increasing amounts (2–12 µg of plasmid transfected) of (B) α_1B_ receptor (red) or β_1_ receptor (blue) or (C) β_1_ receptor (red) or α_1B_ receptor (blue). Both fluorescence and luminescence of each sample were measured prior to every experiment to confirm that there were no changes in the expression of D_4_-RLuc, α_1B_-YFP, or β_1_-YFP constructs. BRET data (see above) are expressed as means ± S.D. of three different experiments. Significant differences with respect to cells not expressing α_1B_ or β_1_ receptors were calculated by one-way ANOVA followed by a Dunnett's multiple comparison post hoc test (*p<0.05 and **p<0.01). (D) Confocal microscopy images of HEK-293T cells transfected with 1 µg of plasmid coding for D_4_-RLuc and 0.5 µg of plasmid coding for α_1B_-YFP or β_1_-YFP. Proteins were identified by fluorescence or by immunocytochemistry. D_4_-RLuc receptor is shown in red, α_1B_-YFP and β_1_-YFP receptors are shown in green, and co-localization is shown in yellow. Scale bar, 5 µm. (E and F) Co-immunoprecipitation of D_4_ and α_1B_ or D_4_ and β_1_ receptors expressed in HEK-293T cells. Membranes from cells transfected with the indicated receptors were solubilized and processed for immunoprecipitation as described under Materials and Methods using goat anti-D_4_R, rabbit anti-α_1_ or goat anti-β_1_ receptor antibodies, or as negative controls (NC), goat anti-adenosine A_2B_ receptor antibody (top in F) or rabbit anti-adenosine A_1_ receptor antibody (bottom in F). Solubilized membranes (E) and immunoprecipitates (F) were analyzed by SDS-PAGE and immunoblotted using rabbit anti-YFP, rabbit anti-α_1_, or goat anti-β_1_ antibody. IP, immunoprecipitation; WB, Western blotting (numbers are included to delineate the different lanes on the SDS-PAGE); MW, molecular mass.

Having shown that D_4_ receptors are functional in the pineal gland, we sought to test whether D_4_ receptors might form heteromers with the adrenergic receptors α_1B_ and β_1_. We first examined this possibility using transfected cells. The best assay for detecting an interaction between two membrane receptors in transfected cells is through biophysical means using Bioluminescence Resonance Energy Transfer (BRET) assays. BRET is particularly useful for testing for complexes with GPCRs as the Förster distance (distance at which the energy transfer efficiency is 50%) of the BRET pairs used here is 4.4 nm, which is 44 angstroms [33]. A single GPCR has a diameter of ∼50 angstroms; thus, the sensitivity and distance requirements of BRET are well suited for working with GPCR complexes. BRET experiments were performed by fusing one of the receptors to the bioluminescent protein *Renilla Luciferase* (RLuc) and the other to a yellow fluorescent protein (YFP) (Materials and Methods). Prior to BRET experiments, preliminary experiments showed that fusion proteins were able to bind their respective ligands with similar affinities (unpublished data). Next, we confirmed that the fusion proteins were able to activate p-ERK 1/2 in the same manner as the native protein (Figure S1) and that all receptors were properly trafficked to the cell membrane as observed by confocal microscopy (Figure 2D). Clear BRET saturation curves were obtained in cells expressing D_4_-RLuc receptors and increasing amounts of α_1B_-YFP or β_1_-YFP receptors (Figure 2A) with BRET_max_ values of 74±4 mBU and 120±10 mBU, respectively, and BRET_50_ values of 37±2 and 61±4, respectively, indicating that the two receptors are indeed forming a higher order structure that allows energy transfer. In contrast, a low and linear BRET was detected in cells expressing α_1B_-RLuc and increasing amounts of β_1_-YFP (Figure 2A, gray line); this was qualitatively similar to the results obtained with the negative control, cells expressing D_4_-RLuc receptors and increasing amounts of D_1_-YFP (Figure 2A, green line). Taken together, these results strongly suggest that the D_4_ receptor forms heteromers with both α_1B_ and β_1_ receptors, but heteromers are not formed between α_1B_ and β_1_ receptors.

Although these results show that α_1B_ and β_1_ do not form heteromers in cells not expressing D_4_ receptors, they do not discount the possibility that there are heterotrimers between D_4_, α_1B_ and β_1_ receptors in cells expressing all three, as has been previously reported for other GPCRs [34]. If α_1B_-β_1_-D_4_ heterotrimers are formed, the molecular determinants on the D_4_ receptor that interact with the α_1B_ receptor must be different from those required to interact with β_1_ receptors. On the other hand, if α_1B_ and β_1_ receptors interact with the same molecular determinants on the D_4_ receptor, α_1B_-β_1_-D_4_ receptor heterotrimers will not form due to the steric hindrance of two receptors competing for the same region. To test this we performed two parallel experiments. In the first one we titrated α_1B_ receptors in cells expressing a constant amount of D_4_-RLuc and α_1B_-YFP (Figure 2B). As more unlabeled α_1B_ was expressed (red line) energy transfer was decreased due to the receptor competing with itself. We observed a nearly identical decrease in energy transfer when we titrated β_1_ receptor. We obtained similar results in the second experiment, when we titrated α_1B_ or β_1_ receptors in cells expressing a constant amount of D_4_-RLuc and β_1_-YFP (Figure 2C). One important observation is that the BRET approached zero as more competing receptor was added, arguing against the possibility that the unlabeled receptor is forming a complex with an existing BRET complex. In the latter scenario, the BRET is likely to remain relatively constant over a range of concentrations of the competing receptor.

The advantage of BRET experiments is that they are performed on live cells in native membranes. However, we sought to confirm these interactions using the classical method of co-immunoprecipitation. We first confirmed that α_1B_ and β_1_ receptors could be co-precipitated with D_4_ receptor. In cells expressing D_4_-YFP, α_1B_ and β_1_ receptors (Figure 2E), immunoprecipitating with anti-D_4_ receptor antibodies led to co-precipitation of both α_1B_ and β_1_ receptors (Figure 2F, lanes 7 and 14). We also performed the reverse, immunoprecipitating with antibodies to α_1B_ or β_1_ receptors and looking for co-precipitation of D_4_ receptor. However, the D_4_ receptor antibodies do not function by Western blot, so we blotted the membrane with an anti-YFP antibody. Immunoprecipitating with either α_1B_ and β_1_ receptor antibodies led to co-precipitation of D_4_ receptors (Figure 2E, lanes 1 and 8). As controls we performed the immunoprecipitation with an unrelated antibody, and no α_1B_ and β_1_ receptors were precipitated (Figure 2F, lanes 6 and 13). Next, to confirm the BRET competition experiments detailed above (Figure 2B and C) we examined the ability of α_1B_ and β_1_ receptors to co-precipitate each other. As can be seen in Figure 2F, lanes 2 and 9, α_1B_ and β_1_ receptors did not co-precipitate. Taken together, these results confirm the BRET experiments and prompted us to discard the possibility of α_1B_-β_1_-D_4_ receptor heterotrimers. Control experiments using cells expressing a single receptor or two receptors were also performed (Figure S2), confirming the above described results.

### Functional Consequences of α_1B_-D_4_ and β_1_-D_4_ Receptor Heteromer Formation in Transfected Cells

**Figure 3:**
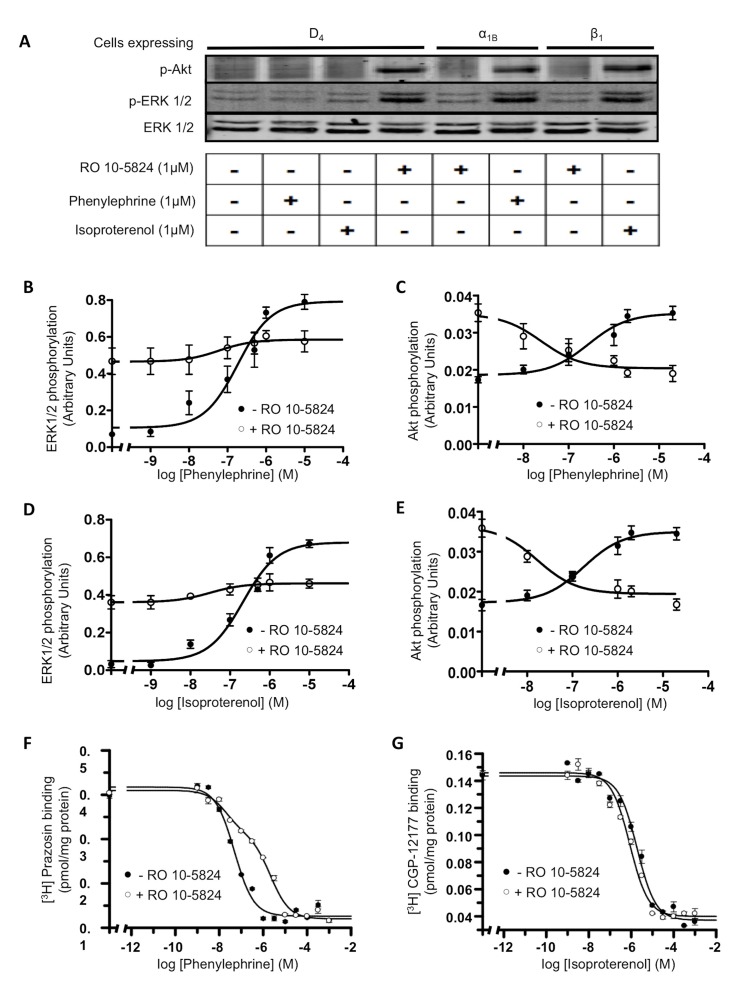
Functional characteristics of α_1B_-D_4_ and β_1_-D_4_ receptor heteromers in transfected cells. CHO cells were transfected with 2 µg of plasmid coding for D_4_ receptors or with 3 µg of plasmid coding for α_1B_ receptors or β_1_ receptors alone (A) or in combination (B to G). In (A), the selectivity of ligands was tested by measuring ERK 1/2 (Thr^183^-Tyr^185^) and Akt (Ser^473^) phosphorylation in cells expressing D_4_, α_1B_, or β_1_ receptors, treated for 7 min with 1 µM RO 10-5824, phenylephrine, or isoproterenol. In (B to E), cells expressing D_4_ and α_1B_ receptors (B and C) or D_4_ and β_1_ receptors (D and E) were treated for 7 min with increasing concentrations of phenylephrine (B and C) or isoproterenol (D and E) in the presence (○) or in the absence (•) of 500 nM RO 10-5824. The immunoreactive bands, corresponding to ERK 1/2 (B and D) and Akt (C and E) phosphorylation of four experiments, were quantified and expressed as mean ± S.E.M. of arbitrary units. In (F and G) membranes of cells expressing D_4_ and α_1B_ receptors (F) or D_4_ and β_1_ receptors (G) were used to perform competition binding experiments of α_1_ receptor antagonist [^3^H]prazosin (1 nM) versus increasing concentrations of phenylephrine (1 nM to 1 mM) (F) or β_1_ receptor antagonist [^3^H]CGP-12177 (1 nM) versus increasing concentrations of isoproterenol (1 nM to 1 mM) (G) in the presence (○) or in the absence (•) of 500 nM RO 10-5824.

A common and often essential attribute of receptor heteromers is the ability to modify downstream signaling versus the single constituent receptors. This type of receptor-receptor interaction has been observed for several receptor heteromers [35]–[38]. To understand the function of α_1B_-D_4_ and β_1_-D_4_ receptor heteromers, we investigated whether there were changes in MAPK (ERK 1/2 phosphorylation) and Akt/PKB (Ser-473 Akt phosphorylation) signaling when heteromers were co-stimulated with both agonists or blocked with antagonists. In terms of pineal function, phosphorylation of ERK 1/2 is the last step in a cascade of signaling that modulates the enzyme that converts 5-HT to N-acetyl serotonin, thus we felt it pertinent to study changes in this signaling pathway. First, the selectivity of receptor agonists, RO 10-5824, phenylephrine, and isoproterenol was tested in cells expressing D_4_, α_1B_, or β_1_ receptors (Figure 3A). Using a selective agonist in time-response assays, we found an increase in ERK 1/2 and Akt/PKB phosphorylation in cells only expressing D_4_, α_1B_, or β_1_ receptors (Figure S3). We next explored whether any cross-talk between the receptors could be detected in cells co-expressing the receptors. In α_1B_-D_4_ and β_1_-D_4_ receptor co-expressing cells, stimulation of D_4_ receptors for 7 min with the D_4_ specific ligand RO 10-5824 inhibited α_1B_ and β_1_ receptor-mediated ERK 1/2 and Akt/PKB activation induced by increasing amounts of phenylephrine and isoproterenol (Figure 3B to E). We observed an almost complete block in the amount of p-ERK 1/2 induced by adrenergic agonists in the presence of RO 10-5824 (Figure 3B and D), indicating that D_4_ activation inhibited the α_1B_ and β_1_ receptor-mediated ERK 1/2 phosphorylation. In addition, a complete block of p-Akt production was observed in the presence of both adrenergic receptor agonist and D_4_ receptor agonist (Figure 3C and E), demonstrating that D_4_ receptor activation inhibited the α_1B_ and β_1_ receptor-mediated Akt/PKB phosphorylation and vice versa. These results are not due to a change in the time in which the signaling peaks, since differences were not observed in time-response curves when co-transfected cells were activated with one or both agonists (Figure S4). In addition, as a negative control, we confirmed that RO 10-5824 did not modify ERK 1/2 or Akt/PKB phosphorylation induced by phenylephrine or isoproterenol in cells transfected with α_1B_ or β_1_ receptors alone (Figure S5).

In addition to cross-talk at the level of receptor signaling, some GPCR heterodimers act at the level of ligand binding [36],[39]–[41]. To explore whether D_4_ receptor ligands can modify the binding of α_1B_ or β_1_ receptor ligands, we performed radioligand competition assays in transfected cells in the presence or absence of the D_4_ receptor specific ligand RO 10-5824. As can be seen in Figure 3F, the addition of RO 10-5824 led to a decrease in the ability of phenylephrine, the α_1B_ receptor agonist, to displace the radiolabeled α_1B_ receptor antagonist [^3^H]-prazosin. The monophasic competition curve giving an affinity constant (K_D1_) of 10±1 nM changed to a biphasic curve giving a K_D1_ of 27±7 nM and K_D2_ of 1,600±400 nM in the presence of RO 10-5824, showing negative cooperativity (cooperativity index of −1.17). These results point out that agonist binding to the D_4_ receptor in the heteromer decreases the affinity of agonist binding to the α_1B_ receptor. Interestingly, when similar experiments were performed testing agonist binding to β_1_ receptors, there were no differences observed in the displacement curve or the affinity in the presence or absence of RO 10-5824 (K_D1_ of 300±50 nM and 460±80 nM, respectively) (Figure 3G). Taken together, these results imply differences between α_1B_-D_4_ and β_1_-D_4_ receptor heteromers in their allosteric interactions.

**Figure 4:**
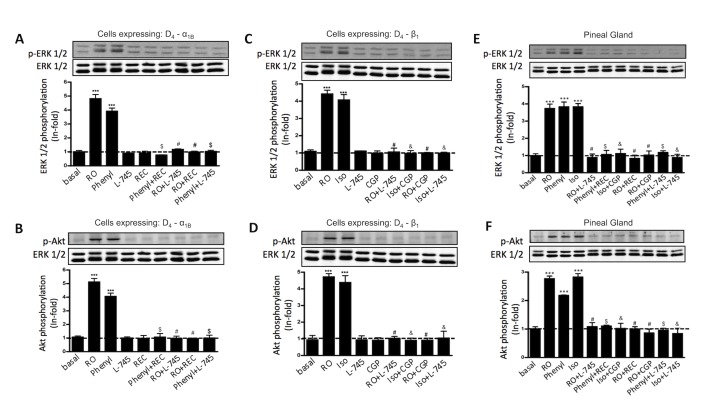
Cross-antagonism between D_4_ and α_1B_ or β_1_ receptors in transfected cells and in pineal gland. In (A to D) CHO cells were transiently co-transfected with 2 µg of plasmid coding for D_4_ receptors and with 3 µg of plasmid coding for α_1B_ receptors (A and B) or β_1_ receptors (C and D). In (E and F) rat pineal glands were extracted at 9:00 h and processed as indicated in Materials and Methods. Cells were treated for 7 min and pineal glands were treated for 10 min with 500 nM of RO 10-5824 (RO), phenylephrine (Phenyl), or isoproterenol (Iso) or with 1 µM of L-745,870 (L-745), REC 15/2615 (REC), or CGP 20712 (CGP), alone or in combination. The immunoreactive bands, corresponding to ERK 1/2 (Thr^183^-Tyr^185^) phosphorylation (A, C, and E) and Akt (Ser^473^) phosphorylation (B, D, and F) of four experiments were quantified and values represent the mean ± S.E.M. of the fold increase with respect to basal levels found in untreated cells. Significant differences were calculated by a one-way ANOVA followed by post hoc Bonferroni's tests (***p<0.001, as compared to the basal level; ^#^ *p*<0.001, as compared to the sample treated with RO 10-5824; ^$^ *p*<0.001, as compared to the sample treated with phenylephrine; ^&^ *p*<0.001, as compared to the sample treated with isoproterenol). A representative Western blot is shown at the top of each panel.

We next looked for a heteromer specific biochemical property. Antagonists, by definition, do not signal; thus, cross-antagonism, any change in α_1B_ or β_1_ mediated signaling caused by an antagonist of D_4_ receptors, could only be due to protein-protein contact between the receptors, and would constitute a specific biochemical characteristic of the heteromer. Prior to looking for cross-antagonism, we investigated the selectivity of D_4_, α_1B_, and β_1_ receptor antagonists by measuring MAPK and Akt/PKB signaling in cells transfected with only D_4_, α_1B_, or β_1_ receptors and stimulated or not with agonist and treated with the selective D_4_, α_1B_, and β_1_ receptor antagonists L-745,870, REC 15/2615, and CGP 20712, respectively. All antagonists behaved as classical antagonists, since none demonstrated any signaling properties in transfected cells (Figure S6). Importantly, all antagonists were selective, as expected, and were able to attenuate agonist-induced signaling in only their respective receptors (Figure S6). Next, cells co-expressing α_1B_-D_4_ and β_1_-D_4_ receptors were treated with antagonists prior to activation with agonist. We obtained a striking cross-antagonism in MAPK and Akt/PKB activation (Figure 4). In both cases, the D_4_ receptor antagonist L-745,870 was able to completely block signaling caused by isoproterenol or phenylephrine. Moreover, signaling induced by the D_4_ receptor agonist was blocked by the adrenergic receptor antagonist REC 15/2615 and CGP 20712. These results demonstrate that the dopamine D_4_ receptor is able to modify α_1B_ and β_1_ function via receptor heteromers and vice versa. In addition, this cross-antagonism constitutes a specific biochemical property of the α_1B_-D_4_ and β_1_-D_4_ receptor heteromers and can be used as a biochemical fingerprint to detect the heteromers in native tissues.

### Functional α_1B_-D_4_ and β_1_-D_4_ Receptor Heteromers in the Pineal Gland

We next sought to detect α_1B_-D_4_ and β_1_-D_4_ receptor heteromers in the pineal gland. We looked for the heteromer biochemical property identified above, the cross-antagonism, as an initial demonstration of the existence of α_1B_-D_4_ and β_1_-D_4_ receptor heteromers in the pineal gland. Therefore, whole pineal glands were isolated 1 h after starting the light period and stimulated with the respective D_4_, α_1B_, and β_1_ agonists RO 10-5824, phenylephrine, and isoproterenol, and p-ERK 1/2 (Figure 4E) and p-Akt (Figure 4F) signaling were measured with respect to basal levels. As can be seen in Figure 4E and F, all three receptors showed robust signaling that could be attenuated with the respective antagonist (L-745,870, REC 15/2615, and CGP 20712). We also detected a cross-antagonism in MAPK and Akt/PKB activation. In both cases, the D_4_ receptor antagonist L-745,870 was able to block completely the signaling caused by isoproterenol or phenylephrine, and the signaling induced by the D_4_ receptor agonist was blocked by the adrenergic receptor antagonist REC 15/2615 and CGP 20712 (Figure 4E and F). These results matched the cross-antagonism observed in transfected cells, thus strongly indicating that D_4_ receptors form functional heteromers with α_1B_ and β_1_ receptors in the pineal gland.

### Direct Detection of α_1B_-D_4_ and β_1_-D_4_ Receptor Heteromers in the Pineal Gland

**Figure 5:**
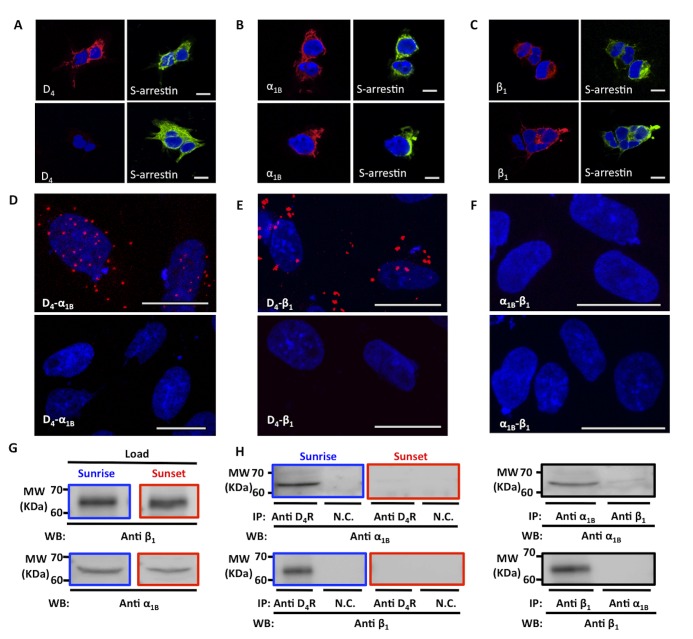
D_4_ receptors form heteromers with α_1B_ and β_1_ receptors in the pineal gland. In (A to C), pinealocytes were isolated from pineal glands extracted at 9:00 h (top) or at 20:00 h (bottom) and stained using anti-S-arrestin antibody (green) and anti-D_4_ (A), anti-α_1B_ (B), or anti-β_1_ (C) antibodies (red) as indicated in Materials and Methods. Scale bar, 5 µm. In (D to F), pinealocytes were isolated from pineal glands extracted at 9:00 h (top) or at 20:00 h (bottom) and the expression of α_1B_-D_4_ (D) and β_1_-D_4_ (E) receptor heteromers was visualized as punctate red fluorescent spots detected by confocal microscopy using the proximity ligation assay (see Materials and Methods). Any expression of α_1B_-β_1_ receptor heteromers was seen (F). Scale bar, 20 µm. In (G and H), co-immunoprecipitation of D_4_ and α_1B_ or D_4_ and β_1_ receptors from pineal gland extracted at 9:00 h (sunrise) or at 20:00 h (sunset) was performed. Glands were solubilized and processed for immunoprecipitation as described under Materials and Methods using goat anti-D_4_, rabbit anti-α_1_, or goat anti-β_1_ receptor antibodies or goat anti-adenosine A_2B_ receptor antibody as a negative control (N.C.). Solubilized gland membranes (G) and immunoprecipitates (H) were analyzed by SDS-PAGE and immunoblotted using rabbit anti-α_1_, rabbit anti-β_1_ receptor antibodies, or goat anti-β_1_ receptor antibody. Immunoprecipitation experiments with anti-α_1_ or anti-β_1_ receptor antibodies (right image in H) were performed with pineal glands extracted at 9:00 h. IP, immunoprecipitation; WB, western blotting; MW, molecular mass.

Biophysical techniques to detect heteromers directly cannot be easily applied in native tissue, but other direct methods can be used. One example is the application of the newly developed proximity ligation assay (PLA). This technique has been successfully employed to detect protein dimers in cells and in tissue [42]. Prior to performing PLA, we first confirmed the antibody specificity. The antibody against D_4_, α_1B_, or β_1_ receptor only stained cells expressing the corresponding receptor but not non-transfected cells, and cells expressing D_4_ receptors were not stained by antibodies against adrenergic receptors, and cells expressing α_1B_ or β_1_ receptors are not stained with anti-D_4_ receptors antibody (Figure S7). The selectivity for anti-D_4_ antibody was also demonstrated by taking advantage of the fact that rat pineal *Drd4 mRNA* expression was found to be circadian in nature, being high at the last part of the dark period and very low during the light period [8],[9]. Thus, without the need of genetically manipulated animals, we observed that the anti-D_4_ antibody was able to stain pinealocytes from pineal glands extracted just after the darkness period but not pinealocytes from glands extracted at the end of the light period (Figure 5A). The expression of both adrenergic receptors was similar in both periods (Figure 5B and C). After testing the expression of the individual receptors using immunofluorescence in pinealocytes, we next looked for evidence of expression of α_1B_-D_4_ and β_1_-D_4_ receptor heteromers in pineal gland using the proximity ligation assay. This direct method requires that both receptors be close enough to allow the two different antibody probes to be able to ligate (<17 nm) [42],[43]. If the receptors are within sufficient proximity, a punctate fluorescent signal can be detected by confocal microscopy (see Materials and Methods). We found that the endogenously expressed D_4_ receptors do indeed form heteromers with the endogenous expressed α_1B_ and β_1_ receptors in a primary culture of pinealocytes obtained from a pineal gland dissected 1 h after the start of the light period (Figure 5D and E, punctate pattern of fluorescence in the upper images), but we did not observe receptor interaction, in the form of a fluorescent signal, for negative controls tested in the absence of primary antibodies (Figure S8) or for α_1B_-β_1_ receptors (Figure 5F). These results were consistent with the BRET experiments and demonstrated α_1B_-D_4_ and β_1_-D_4_ receptor heteromers expression in pinealocytes. As we observed a severe depletion of D_4_ receptor expression in pinealocytes from glands isolated at the end of the light period, we performed the PLA experiments also with glands isolated at the end of the light period. As expected, no α_1B_-D_4_ and β_1_-D_4_ receptor heteromers were detected (Figure 5D and E, lower images). These results not only confirm the specificity of the results in Figure 5D and E (top images), but also demonstrate the circadian nature of heteromer formation. To confirm the circadian nature of heteromer formation we performed co-immunoprecipitation experiments using glands dissected 1 h after the start of the light period (sunrise) or glands isolated at the end of the light period (sunset). Although adrenergic receptors are expressed in sunrise and sunset periods (Figure 5G), immunoprecipitating with anti-D_4_ receptor antibodies led to co-precipitation of both α_1B_ and β_1_ receptors only from glands extracted at the sunrise period and not from glands extracted at the sunset period (Figure 5H), indicating the heteromer expression in the pineal gland and the circadian nature of the heteromerization. The lack of heteromer formation between α_1B_ and β_1_ receptors seen earlier by BRET and immunoprecipitation in transfected cells was confirmed in pineal gland by co-immunoprecipitation experiments (Figure 5H).

### Functional Consequences of α_1B_-D_4_ and β_1_-D_4_ Receptor Heteromer Formation in the Pineal Gland

**Figure 6:**
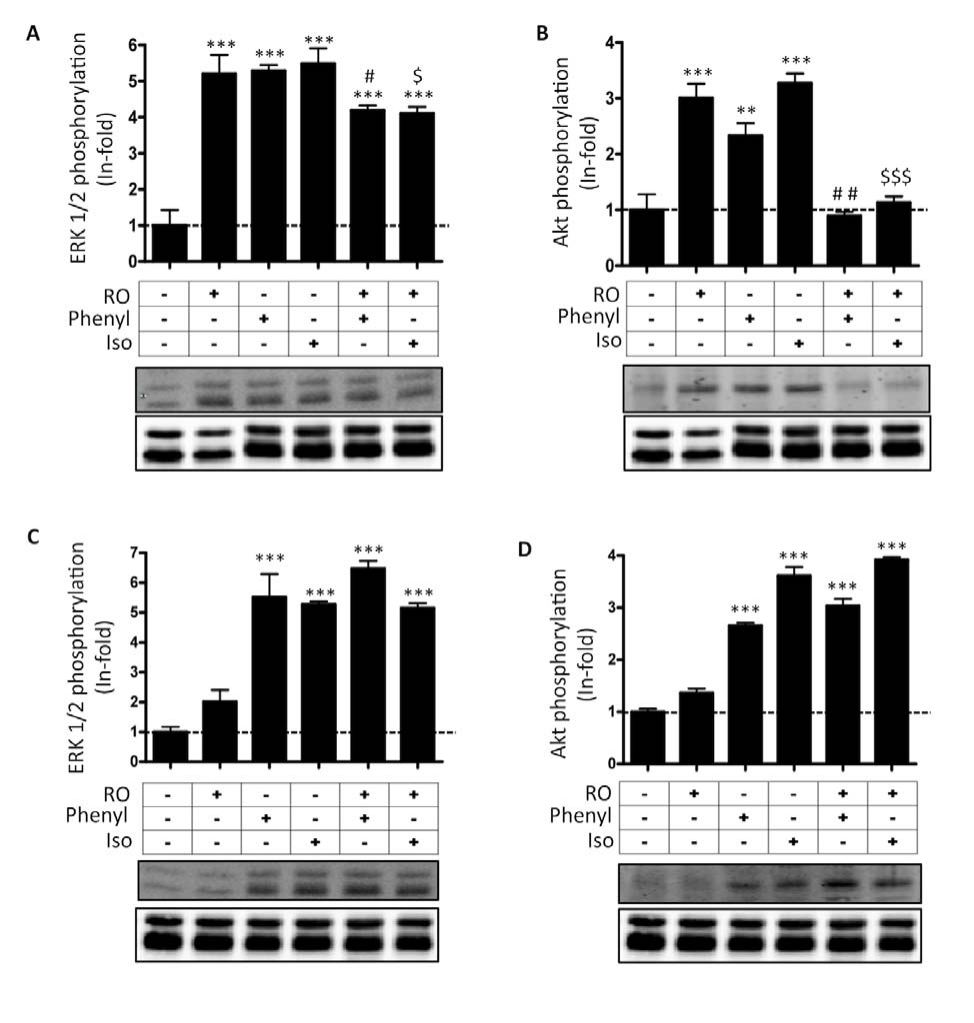
Functional characteristics of α_1B_-D_4_ and β_1_-D_4_ receptor heteromers in pineal gland. Pineal glands extracted at 9:00 h (A and B) or at 20:00 h (C and D) were treated for 10 min with RO 10-5824 (RO), phenylephrine (Phenyl), or isoproterenol (Iso) at 1 µM concentration alone or in combination. The immunoreactive bands, corresponding to ERK 1/2 (Thr^183^-Tyr^185^) (A and C) or Akt (Ser^473^) (B and D) phosphorylation, of three experiments performed in duplicates were quantified, and values represent the mean ± S.E.M. of the fold increase with respect to basal levels found in untreated pineal glands. Significant differences were calculated by a one-way ANOVA followed by post hoc Bonferroni's tests (**p<0.01 and ***p<0.001, as compared to the basal level. ^#^ *p*<0.05 and ^##^ *p*<0.01, as compared to the sample treated with phenylephrine; ^$^ *p*<0.05 and ^$$$^ *p*<0.001, as compared to the sample treated with isoproterenol). A representative Western blot is shown at the bottom of each panel.

To test the effect of receptor co-activation in α_1B_-D_4_ and β_1_-D_4_ receptor heteromers in the p-ERK 1/2 and p-Akt/PKB production, pineal glands, isolated at 9:00 h, 1 h after the start of the light period (at sunrise), were stimulated with RO 10-5824, phenylephrine, or isoproterenol alone or in combination. Co-activation with RO 10-5824 and phenylephrine or with RO 10-5824 and isoproterenol induced a significant decrease of p-ERK 1/2 production compared with stimulation with one agonist alone (Figure 6A). Co-activation completely blocked the formation of p-Akt/PKB in cells stimulated with RO 10-5824, phenylephrine, or isoproterenol (Figure 6B). These results indicate that there is a negative cross-talk between D_4_ and α_1B_ or β_1_ receptors not only in transfected cells but also in the pineal gland. To be sure that the data reflected a true negative cross-talk between D_4_ and α_1B_ or β_1_ receptors, and not a time displacement of the signaling, we performed time-response experiments with pineal glands (Figure S9). The effect of co-activation with RO 10-5824 and phenylephrine or with RO 10-5824 and isoproterenol on α_1B_ and β_1_ signaling was not due to a change in timing of the signal, with maximal signal obtained at 10 min. In addition, at all times examined no p-Akt/PKB signal was detected in the presence of both adrenergic agonists and RO 10-5824. These data support the conclusion that the results observed in Figure 6A and B were indeed due to a true negative cross-talk.

As the expression of D_4_ receptor in the pineal gland is regulated by a cycle of light/dark, we reasoned that if we isolated pineal gland after 12 h of light (at sunset) when the levels of D_4_ receptor are low, then we should now lose the negative cross-talk seen in Figure 6A and B. To test this, we stimulated pineal gland extracted at 20:00 h and compared signaling after stimulation with RO 10-5824 in the presence or absence of phenylephrine and isoproterenol. As shown in Figure 6C and D, there was no inhibition of α_1B_ and β_1_ receptor-mediated MAPK and Akt/PKB activation by the D_4_ receptor agonist RO 10-5824 in glands isolated at the end of the light period (sunset), a time of low D_4_ receptors expression. This was in contrast to signaling in glands extracted at 9:00 h, just after the dark period (sunrise) where D4 receptors are expressed and negative cross-talk in agonist-induced signaling was observed (Figure 6A and B).

### The Metabolic Consequences of α_1B_-D_4_ and β_1_-D_4_ Receptor Heteromers Activation in the Pineal Gland

Finally, we sought to understand how α_1B_-D_4_ and β_1_-D_4_ receptor heteromers might modulate pineal gland function. A major role of the pineal gland is controlling the levels of melatonin and its precursor 5-HT via synthesis and release. The α_1B_ receptor controls 5-HT and melatonin release via potentiation of the calcium-induced exocytosis, while the β_1_ receptors can modify the synthesis of both 5-HT and melatonin [15]–[18]. With this in mind, we tested the role of the α_1B_-D_4_ and β_1_-D_4_ receptor heteromers in 5-HT and melatonin synthesis and release. Ideally, to test the physiological importance of heteromers, one would like to create a targeted knockout animal lacking one of the partner receptors in the tissue of interest to be compared with wild type animals. However, in the case of D_4_ receptor expression in the pineal gland, nature provided a suitable alternative. We decided to take advantage of the fact that D_4_ receptor expression is altered by the cycle of light and dark and compare results obtained with pineal gland extracted at the end of the light period (sunset), when D_4_ receptors are not expressed, with those obtained with glands extracted at the end of the dark period (sunrise), when D_4_ receptors are expressed.

**Figure 7:**
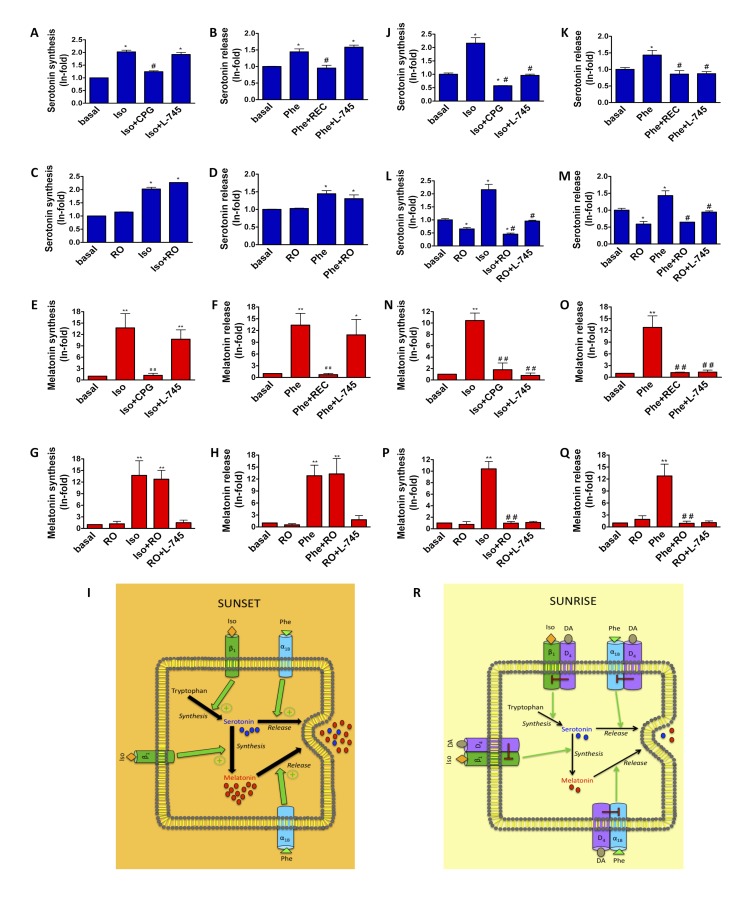
Metabolic consequences of α_1B_-D_4_ and β_1_-D_4_ receptor heteromers activation. 5-HT synthesis (A, C, J, L) and release (B, D, K, M) and melatonin synthesis (E, G, N, P) and release (F, H, O, Q) were measured as described in Materials and Methods in pineal gland extracted at 20:00 h (A to H) or at 9:00 h (J to Q). Pineal glands were not treated (basal) or treated with 500 nM RO 10-5824 (RO), 500 nM phenylephrine (Phe), 500 nM isoproterenol (Iso), 1 µM L-745,870 (L-745), 1 µM REC 15/2615 (REC), or 1 µM CGP 20712 (CGP), alone or in combination. Three experiments were quantified and values represent the mean ± S.E.M. of the fold increase with respect to basal levels found in untreated pineal glands. Significant differences were calculated by a one-way ANOVA followed by post hoc Bonferroni's tests (*p<0.01 as compared to the basal level; ^#^ *p*<0.005 as compared to the sample treated with isoproterenol or with phenylephrine). In (I and R) the overall results are presented as a scheme.

We treated pineal glands, isolated at 20:00 h, when α_1B_-D_4_ and β_1_-D_4_ receptor heteromers are not expressed, with specific agonists and/or antagonists and measured the amount of 5-HT synthesized (Figure 7A and C) or released (Figure 7B and D) and the amount of melatonin synthesized (Figure 7E and G) or released (Figure 7F and H). As can be seen in Figure 7A to H, treatment with the D_4_-specific agonist, RO 10-5824 showed no increase in either 5-HT or melatonin synthesis or release compared to basal levels. In contrast, we observed a large increase in melatonin synthesis and release when the glands were treated with the β_1_ receptor agonist isoproterenol or the α_1B_ agonist phenylephrine, respectively (Figure 7E to H), and a significant increase in 5-HT synthesis and release when the glands were treated with isoproterenol or phenylephrine (Figure 7A to D). The increases in 5-HT and melatonin synthesis and release could be blocked only by the corresponding specific antagonists of adrenergic receptors but not by the D_4_ receptor antagonist L-745,870 (Figure 7A, B, E, and F), demonstrating a lack of cross-antagonism due to the absence of the heteromers. In addition, when we treated the glands with either phenylephrine or isoproterenol in the presence of the dopamine D_4_ receptor agonist RO 10-5824 (Figure 7C, D, G, and H), no negative cross-talk between dopamine D_4_ and adrenergic receptors could be detected. The role of adrenergic receptors is represented in Figure 7I. In contrast and very interestingly, when pineal glands were isolated at 9:00 h, at sunrise (when D_4_ receptor expression increases and α_1B_-D_4_ and β_1_-D_4_ receptor heteromers are expressed) and were stimulated as before with agonists of both α_1B_ and β_1_ receptors in the presence of either the pertinent antagonist or the D_4_ antagonist, we observed that 5-HT and melatonin synthesis and release could be blocked not only by the corresponding specific antagonists of adrenergic receptors but also by the D_4_ receptor antagonist L-745,870 (Figure 7J, K, N, and O). This demonstrates a clear cross-antagonism. In addition, when we treated the glands with either phenylephrine or isoproterenol in the presence of the dopamine D_4_ receptor agonist RO 10-5824, a complete block in the ability of either ligand to increase 5-HT or melatonin synthesis or release was observed (Figure 7L, M, P, and Q). This shows that, in these conditions, a negative cross-talk between dopamine D_4_ and adrenergic receptors exists in the pineal gland. A schematic representing the influence of dopamine on 5-HT and melatonin synthesis and release in these conditions is represented in Figure 7R. These data provide strong evidence that the role of the dopamine D_4_ receptor via either α_1B_-D_4_ and β_1_-D_4_ receptor heteromers is to modify the melatonin metabolic pathway in the pineal gland.

## Discussion

In the present study we identified a previously unknown mechanism for how dopamine can regulate adrenergic receptor function in a circadian fashion. By applying a number of different experimental approaches, we were able to identify (1) that functional dopamine D_4_ receptors form heteromers with both α_1B_- and β_1_ adrenergic receptors in transfected cells and in the pineal gland; (2) that the α_1B_-D_4_ and β_1_-D_4_ receptor heteromers allow direct modulation of the adrenergic agonist-induced MAPK and Akt signaling by the D_4_ receptor agonist and antagonist in transfected cells and in the pineal gland; (3) that the synthesis of melatonin and its precursor 5-HT, promoted by adrenergic receptor stimulation in the pineal gland, can be controlled by D_4_ receptor activation via α_1B_-D_4_ and β_1_-D_4_ receptor heteromers; and (4) that this D_4_ receptor heteromer-mediated modulation is dependent on the circadian light/dark cycle. This is the first example, to our knowledge, of a circadian-dependent modulation of receptor heteromerization. Together these findings point to a new role for the D_4_ receptor in the pineal gland where D_4_ receptor activation modifies α_1B_- and β_1_ adrenergic receptor function by a direct receptor-receptor interaction, which can limit the levels of melatonin secreted by the pineal gland.

The adrenergic receptors are the mainstay receptors of pineal gland function. They form the bridge between the circadian controlled release of norepinephrine by the surrounding sympathetic nerve terminals and the melatonin production of the pineal gland. The adrenergic receptors are thought to control the production of melatonin through a variety of mechanisms, including control of the levels of the melatonin precursor 5-HT [15],[16]. Dopamine is also present in the afferent sympathetic nerves in the pineal gland, not only as a precursor of norepinephrine, but is also proposed to be co-released to a lesser extent with norepinephrine [8].

The “receptor heteromer” concept, in which receptors of the same and different gene families can combine among themselves to generate new and unique biochemical and functional characteristics, is becoming widely accepted for GPCRs and constitutes an emerging area in the field of GPCR signaling and function regulation [30]. To date there had been no demonstration of heteromers involving dopamine and the adrenergic receptors. Here, by means of BRET experiments in transfected cells and by proximity ligation assays in pinealocytes, we present direct evidence that the D_4_ receptor forms heteromers with both the α_1B_ and β_1_ adrenergic receptors. The formation of α_1B_-D_4_ and β_1_-D_4_ receptor heteromers in the pineal gland manifests itself in the form of cross-antagonism. We observed that a D_4_ receptor-specific antagonist was able to block the signaling through both α_1B_- and β_1_ adrenergic receptors. The specific antagonists of α_1B_- and β_1_ adrenergic receptors were also able to block signaling through D_4_ receptors. This is a clear example of cross-antagonism in a receptor heteromer [44]–[46], since by definition an antagonist is not able to induce intracellular signaling. This statement is *a propos* in our case since none of the antagonists used here demonstrated any signaling activity. Thus the simplest way to explain the effect of a D_4_ receptor antagonist on α_1B_ and β_1_ receptor activation and vice versa is through a direct protein-protein interaction between both receptors.

The functional consequences of this protein-protein interaction is a negative cross-talk between both receptors in the α_1B_-D_4_ and β_1_-D_4_ receptor heteromers—that is, the block in the amount of p-ERK 1/2 induced by adrenergic agonists in the presence of D_4_ receptor agonist and the complete block of p-Akt production when both receptors in the heteromer were co-stimulated. In the pineal gland, D_4_ receptor mRNA expression is tightly regulated so that it is highest during the last part of the dark period [8]. Accordingly, we show that the D_4_ receptor is expressed and is functional in pineal glands isolated at sunrise and we saw no activity and no expression when pineal glands were isolated at sunset, the end of the light period. Our finding that the D_4_ receptor can modify the downstream signaling of the α_1B_ and β_1_ adrenergic receptors is particularly interesting as D_4_ receptor expression was found to be modified by an increase in norepinephrine levels [8]. Norepinephrine levels are also known to increase at night, and it is through its binding to the adrenergic receptors that the level of D_4_ receptor mRNA is thought to reach the maximum at the end of the dark period [8]. Thus, the mechanism we describe may represent a feedback inhibition, where increased expression of D_4_ receptor via adrenergic signaling leads to an increase of α_1B_-D_4_ and β_1_-D_4_ receptor heteromers, which then inhibit adrenergic-induced signaling through the above described cross-talk. The detailed molecular mechanism for how this feedback occurs is less clear. It is known that heteromers can function in a variety of different mechanisms, including allosterically, asymmetrically, and/or through cooperativity [23],[29],[47]. The binding experiments in transfected cells suggest, at least for the α_1B_-D_4_ receptor heteromer, there is inhibition at the level of ligand binding. Why is this not seen for β_1_-D_4_ receptor heteromer? Does this reflect differences in heteromer plasticity—for example, protomer-protomer molecular interactions promoted by ligand binding to one protomer inducing structural changes in the other protomer that are sensed at both ligand binding and signaling levels in one case and only at the signaling level in the other case? Or are these results due to something experimentally related—for example, the differences of ligands used? More experiments will be required to identify how exactly these particular heteromers function. An interesting corollary to heteromer function and the data presented here is that a recent proposal arguing against the existence of heteromers and heteromer function suggested that GPCRs were competing for available G-proteins and that any cross-signaling effects observed were due to this competition [48]. Our results argue against this possibility, at least in the case of α_1B_-D_4_ and β_1_-D_4_ receptor heteromers, as none of these receptors use the same G-proteins to signal. We have observed cross-talk at the level of p-ERK 1/2 and p-Akt, two steps along the production and release of melatonin. Separately all three receptors studied can activate both signaling pathways; thus, heteromer formation by a protein-protein interaction clearly alters the ability of these receptors to signal using these pathways. Cross-talk has been observed for other heteromers [24],, and the mechanisms have varied from changes in β-arrestin recruitment, changes in receptor trafficking, changes in G-protein coupling, and/or changes in ligand binding. More experiments will be required to understand the molecular mechanism at play in the pineal gland. Another possible interpretation of our signaling results is that some downstream effect after D_4_ receptor activation might cause adrenergic receptor signaling to be inhibited. This does not seem to be the mechanism of action for the α_1B_-D_4_ heteromers based on the fact that the inhibition occurs at the binding level as well. However, although we cannot completely rule out this possibility for β_1_-D_4_, the fact that there is cross-antagonism and that the receptors are in a complex suggests an indirect mechanism of inhibition is less likely.

We have also studied the metabolic consequences of α_1B_-D_4_ and β_1_-D_4_ receptor heteromer activation at the level of melatonin synthesis and release, as well as the precursor of melatonin, 5-HT. Melatonin levels are increased at night while 5-HT levels fluctuate in the opposite manner, with production and secretion increasing during the day. Through mass action, large changes in AANAT activity at night, the enzyme in the last step to melatonin synthesis, can rapidly decrease the levels of 5-HT, yielding large increases in melatonin [50]. It is important to point out that 5-HT synthesis is thought to occur both during the day and at night, and nocturnal synthesis and release of 5-HT is required for maximal adrenergic stimulation of melatonin synthesis [51],[52]. Extracellular 5-HT is either taken up by surrounding sympathetic nerves or binds 5HT_2C_ receptors on the pineal gland, which in turn can lead to increased melatonin synthesis and release [51],[53]. To date it has not been entirely clear what limits the maximum nighttime and minimum daytime rates of melatonin production. Our data suggest that α_1B_-D_4_ and β_1_-D_4_ receptor heteromers may play an important role in this process. In pineal glands, isolated at the end of the light period (sunset) when the expression of D_4_ receptor is negligible, treated with adrenergic ligands, we have seen a large increase in melatonin and a moderate increase in 5-HT synthesis mediated by β_1_ receptors and release mediated by α_1B_ receptors (Figure 7I). In this case neither synthesis nor release of 5-HT or melatonin was blocked by treating the gland simultaneously with a D_4_ receptor agonist or was modified in the presence of D_4_ receptor antagonist. In these conditions the pineal gland is starting the melatonin production during the dark period. In pineal glands, isolated at the end of the dark period (sunrise) when the D_4_ receptor is expressed, treated with adrenergic ligands, we have also seen a large increase in melatonin and 5-HT synthesis mediated by β_1_ receptors and release mediated by α_1B_ receptors. Interestingly, both synthesis and release were blocked by treating the gland simultaneously with a D_4_ receptor agonist (Figure 7). Thus, dopamine appears to be able to regulate the melatonin and 5-HT levels as seen in Figure 7R. This suggests that dopamine, via α_1B_-D_4_ and β_1_-D_4_ receptor heteromers, may serve both as a buffer to control the amount of 5-HT that can be made and released during the light period, limiting total melatonin production, and be partially responsible for the block of melatonin production after the dark period. During the day, D_4_ receptors would begin to be down-regulated, less α_1B_-D_4_ and β_1_-D_4_ receptor heteromers would be formed, AANAT would be also down-regulated, maintaining a reduced melatonin production, 5-HT levels would gradually increase, and the cycle could repeat. These findings provide the first report of a role for the D_4_ receptor in the pineal gland and suggest a new area of research on how dopamine receptors, by means of a circadian-related heteromerization with adrenergic receptors, may help maintain the circadian rhythm signals emulating from the pineal gland.

## Materials and Methods

### Fusion Proteins and Expression Vectors

The cDNA for human dopamine D_4_ and adrenergic α_1B_ and β_1_ receptor genes expressed in the *pcDNA3.1* vector were amplified without its stop codon using sense and antisense primers to be cloned in the mammalian humanized pRluc-N1 or in the EYFP-N3 vectors (see Text S1).

### Cell Culture and Transient Transfection

CHO or HEK-293T cells were grown in supplemented α-MEM or Dulbecco's modified Eagle's medium (DMEM) medium, respectively, and they were transfected by the polyethylenimine (PEI) method (see Text S1).

### Immunostaining

HEK-293T cells were grown on glass coverslips and transiently transfected. After 48 h of transfection, cells were fixed and labeled with the corresponding antibodies (see Text S1).

### BRET Assay

HEK-293T cells were co-transfected with a constant amount of cDNA encoding for the receptor fused to Rluc and with increasing amounts of cDNA encoding to the receptor fused to YFP to measure BRET. BRET was expressed as milli BRET Units (mBU) and is the BRET ratio×1,000 (see Text S1).

### Pineal Gland Dissection and Culture

Male Sprague Dawley rats (3-mo-old, ≈350 g), receiving water and food ad libitum, were obtained from the animal facility of the Faculty of Biology (University of Barcelona). Rats were housed in light∶dark 12∶12 lighting cycles starting light at 8:00 h and dark at 20:00 h, and 4% Isoflurane (2-chloro-2-(difluoromethoxy)-1,1,1-trifluoro-ethane) anesthetized animals were killed by decapitation at 9:00 h (just after the dark period) or at 20:00 h (after light period) and pineal glands were immediately dissected. All procedures were approved by the Catalan Ethical Committee for Animal Use (CEAA/DMAH 4049 and 5664). Rat pineal glands were cultured in defined culture medium (BGJb, Invitrogen, Carlsbad, CA) containing 10% (v/v) fetal bovine serum (heat-inactivated) for 24–36 h and cultured in serum-free medium for 16 h before the addition of agonists and/or antagonists for signaling experiments or were overnight cultured in HBSS medium (137 mM NaCl, 5 mM KCl, 0.34 mM Na_2_HPO_4_.12H_2_O, 0.44 mM KH_2_PO_4_, 1.26 mM CaCl_2_.2H_2_O, 0.4 mM MgSO_4_.7H_2_O, 0.5 mM MgCl_2_, 10 mM HEPES, pH 7.4), supplemented with 0.1% glucose, 100 U/ml penicillin/streptomycin, and 1 mg/ml bovine serum albumin, containing agonist and/or antagonist for serotonin synthesis and release determination.

### Coimmunoprecipitation

Transfected cells or pineal glands were solubilized by homogenization in ice-cold immunoprecipitation buffer and were processed for immunoprecipitation as described in the immunoprecipitation protocol using a Dynabeads Protein G kit (Invitrogen) (see Text S1).

### Detection of MAPK and Akt/PKB Phosphorylation

Transfected cells or pineal glands were cultured in serum-free medium before the addition of the indicated concentration of ligands for the indicated time. Both cells and pineal glands were washed and lysed. Proteins were separated by electrophoresis and ERK 1/2 (Thr^183^-Tyr^185^) and Akt (Ser^473^) phosphorylation was determined by Western blot and band densities were quantified (see Text S1).

### Radioligand Binding Experiments

Competition experiments were performed using membranes from cells expressing D_4_ and α_1B_ or β_1_ receptors. Membranes were incubated with the indicated concentration of the α_1B_ receptor antagonist [^3^H]prazosin or β_1_ receptor antagonist [^3^H]CGP-12177 (PerkinElmer Life and Analytical Sciences) and increasing concentrations of phenylephrine or isoproterenol, respectively, in the absence or in the presence of the indicated concentration of the D_4_ receptor agonist RO 10-5824 (Tocris, Aronmouth, UK) (see Text S1).

### Pinealocyte Culture, Signaling, and Immunocytochemistry

Pinealocytes were prepared from rat pineal glands as previously described by Silveira Cruz-Machado et al. [54] and maintained in culture no more than 48 h. For signaling experiments, pinealocytes were treated with specific agonist, fixed with paraformaldehyde, and treated with the corresponding antibodies (see Text S1).

### In Situ Proximity Ligation Assay (PLA)

The primary cultures of pinealocytes were fixed and permeabilized as described above. The receptor-receptor molecular interaction was detected using the Duolink II in situ PLA Detection Kit (see Text S1).

### Serotonin Synthesis and Release Determination

After 36 h of culture in BGJb medium, pineal glands were incubated in supplemented HBSS medium for 12 h with specific agonist and/or antagonist and radioactive [^14^C]-Tryptophan (10 µM). Medium and pineal glands were collected separately and [^14^C]-serotonin in medium or in homogenized glands was separated by HPLC chromatography coupled to detection by fluorescence and counted in a liquid scintillation counter (see Text S1).

### Melatonin Synthesis and Release Determination

After 36 h of culture in BGJb medium, the pineal glands were incubated for 12 h with specific agonist and/or antagonist in supplemented HBSS medium. After incubation, media were collected into eppendorf tubes and pineal glands were homogenized by sonication in a Dynatech/Sonic Dismembrator (Dynatech Labs, Chantilly, VA) for 15 s. An aliquot was reserved for protein quantification by the Lowry method, and cellular debris was removed by centrifugation at 10,000 g for 10 min at 4°C. Melatonin was quantified using a radioimmunoassay kit with [^125^I]-melatonin (DiaSource, Belgium) following the instructions of the supplier.

## Supporting Information

Figure S1 Functionality of the fusion proteins. HEK 293T cells were transfected with 2 µg of plasmid coding for the D_4_ receptor or with 3 µg of plasmid coding for the adrenergic α_1B_ or β_1_ receptors or to the corresponding fusion proteins D_4_-RLuc, α_1B_-YFP, α_1B_-RLuc, or β_1_-YFP. 48 h post-transfection, cells expressing D_4_ or D_4_-RLuc receptors were treated with 500 nM RO 10-5824, cells expressing α_1B_, α_1B_-YFP or α_1B_-RLuc receptors were treated with 1 µM phenylephrine, or cells expressing β_1_ or β_1_-YFP were treated with 1 µM isoproterenol for 7 min and ERK 1/2 (Thr^183^-Tyr^185^) phosphorylation was determined. The immunoreactive bands of three experiments performed in duplicates were quantified and expressed as mean ± S.E.M. of arbitrary units. A representative Western blot is shown at the top. Significant differences with respect to basal levels were calculated by one-way ANOVA followed by a Dunnett's multiple comparison post hoc test (**p<0.01 and ***p<0.001).
(TIF)

Figure S2 Specificity of the antibodies used for co-immunoprecipitation experiments. Membranes from cells expressing the indicated receptors were solubilized and processed for immunoprecipitation as described under Materials and Methods using goat anti-D_4_ or rabbit anti-α_1_ receptor antibodies or goat anti-adenosine A_2B_ or rabbit anti-adenosine A_1_ receptor antibodies as negative controls. Solubilized membranes (Load) and immunoprecipitates were analyzed by SDS-PAGE and immunoblotted using rabbit anti-YFP, rabbit anti-α_1_, rabbit anti-β_1_, or goat anti-β_1_ receptor antibodies. IP, immunoprecipitation; WB, Western blotting; MW, molecular mass.
(TIF)

Figure S3 ERK 1/2 and Akt phosphorylation in cells transfected with D_4_, α_1B_, or β_1_ receptors. CHO cells were transfected with 2 µg of plasmid coding for the D_4_ receptor (A, D), 3 µg of plasmid coding for the α_1B_ receptor (B, E), or 3 µg of plasmid coding for the β_1_ receptor (C, F). 48 h post-transfection, cells were treated for increasing time with 500 nM RO 10-5824 (A, D), 1 µM phenylephrine (B, E), or 1 µM isoproterenol (C, F). The immunoreactive bands, corresponding to ERK 1/2 (Thr^183^-Tyr^185^) (A to C) and Akt (Ser^473^) (D to F) phosphorylation, of three experiments were quantified and expressed as mean ± S.E.M of arbitrary units. Statistical differences over non-treated cells were determined by one-way ANOVA followed by a Dunnett's multiple comparison post hoc test (*p<0.05, **p<0.01, and ***p<0.001).
(TIF)

Figure S4 Time-response on ERK 1/2 and Akt phosphorylation by co-activation of α_1B_-D_4_ and β_1_-D_4_ receptor heteromers in cell cultures. CHO cells were transfected with 2 µg of plasmid coding for the D_4_ receptor and 3 µg of plasmid coding for the α_1B_ receptor (A) or the β_1_ receptor (B). 48 h post-transfection, cells were treated with 1 µM phenylephrine (Phenyl, A) or 1 µM isoproterenol (Iso, B) alone or in the presence of 1 µM RO 10-5824 for different times. A representative Western blot is shown.
(TIF)

Figure S5 Selectivity of D_4_, α_1B_, or β_1_ receptor agonists. The selectivity of ligands was tested by measuring ERK 1/2 (Thr^183^-Tyr^185^) (A) and Akt (Ser^473^) (B) phosphorylation in cells expressing D_4_, α_1B_, or β_1_ receptors, treated for 7 min with 1 µM RO 10-5824 (RO), phenylephrine (Phe), or isoproterenol (Iso) alone or in combination as indicated.
(TIF)

Figure S6 Selectivity of D_4_, α_1B_, or β_1_ receptor antagonists. CHO cells were transfected with 2 µg of plasmid coding for the D_4_ receptor or with 3 µg of plasmid coding for α_1B_ or β_1_ receptors. 48 h post-transfection, cells were treated for 7 min with 500 nM RO 10-5824 (RO), 500 nM phenylephrine (Phenyl), 500 nM isoproterenol (Iso), 1 µM L-745,870 (L-745), 1 µM REC 15/2615 (REC), or 1 µM CGP 20712 (CGP) alone or in combination. The immunoreactive bands, corresponding to ERK 1/2 (Thr^183^-Tyr^185^) (A) and Akt (Ser^473^) (B) phosphorylation, of three experiments were quantified and values represent the mean ± S.E.M. of the fold increase over basal levels found in untreated cells (basal). Significant differences over basal levels were determined by one-way ANOVA followed by a Dunnett's multiple comparison post hoc test (*p<0.05, ***p<0.001). A representative Western blot is shown at the top.
(TIF)

Figure S7 Specificity of the antibodies tested by immunocytochemistry. In (A) non-transfected HEK-293T cells (right panels) and cells transfected with, top to bottom, 1 µg of plasmid coding for D_4_ receptor, 0.5 µg cDNA coding for α_1B_ receptor, or 0.5 µg cDNA coding for β_1_ receptor (left panels) were stained using, top to bottom, anti-D_4_, anti-α_1_, or anti-β_1_ antibodies as indicated in Materials and Methods. Scale bar, 5 µm. In (B to J), cells were transfected with 1 µg of plasmid coding for D_4_-YFP receptor (B to D), 0,5 µg cDNA coding for α_1B_-YFP receptor (E to G), or 0.5 µg cDNA coding for β_1_-YFP receptor (H to J). The expression of the receptors was detected by its own YFP fluorescence (B, E, and H) or by using anti-α_1_ (C and J), anti-β_1_ (D and G), or anti-D_4_ (F and I) receptor antibodies. Scale bar, 5 µm.
(TIF)

Figure S8 Negative controls for in situ proximity ligation assays. Negative controls for in situ proximity ligation assays (PLA, see Materials and Methods) are shown demonstrating a lack of punctate red fluorescence staining in pinealocytes in the absence of primary antibodies, left to right, anti-D_4_, anti-α_1_, or anti-β_1_ antibodies. Scale bar, 20 µm.
(TIF)

Figure S9 Time-response on ERK 1/2 and Akt phosphorylation by co-activation of α_1B_-D_4_ and β_1_-D_4_ receptor heteromers in pineal gland. Pineal glands extracted at 9:00 h were treated with 1 µM phenylephrine (Phenyl) or 1 µM isoproterenol (Iso) in the presence of 1 µM RO 10-5824 for the times indicated. A representative Western blot is shown.
(TIF)

Text S1 Additional details on materials and methods used throughout the article.
(DOC)
