# Supplementary figures and images for: Circadian-Related Heteromerization of Adrenergic and Dopamine D4 Receptors Modulates Melatonin Synthesis and Release in the Pineal Gland
Source: PLoS Biol. 2012 Jun 19;10(6):e1001347. doi: 10.1371/journal.pbio.1001347 (PMC3378626; doi:10.1371/journal.pbio.1001347)

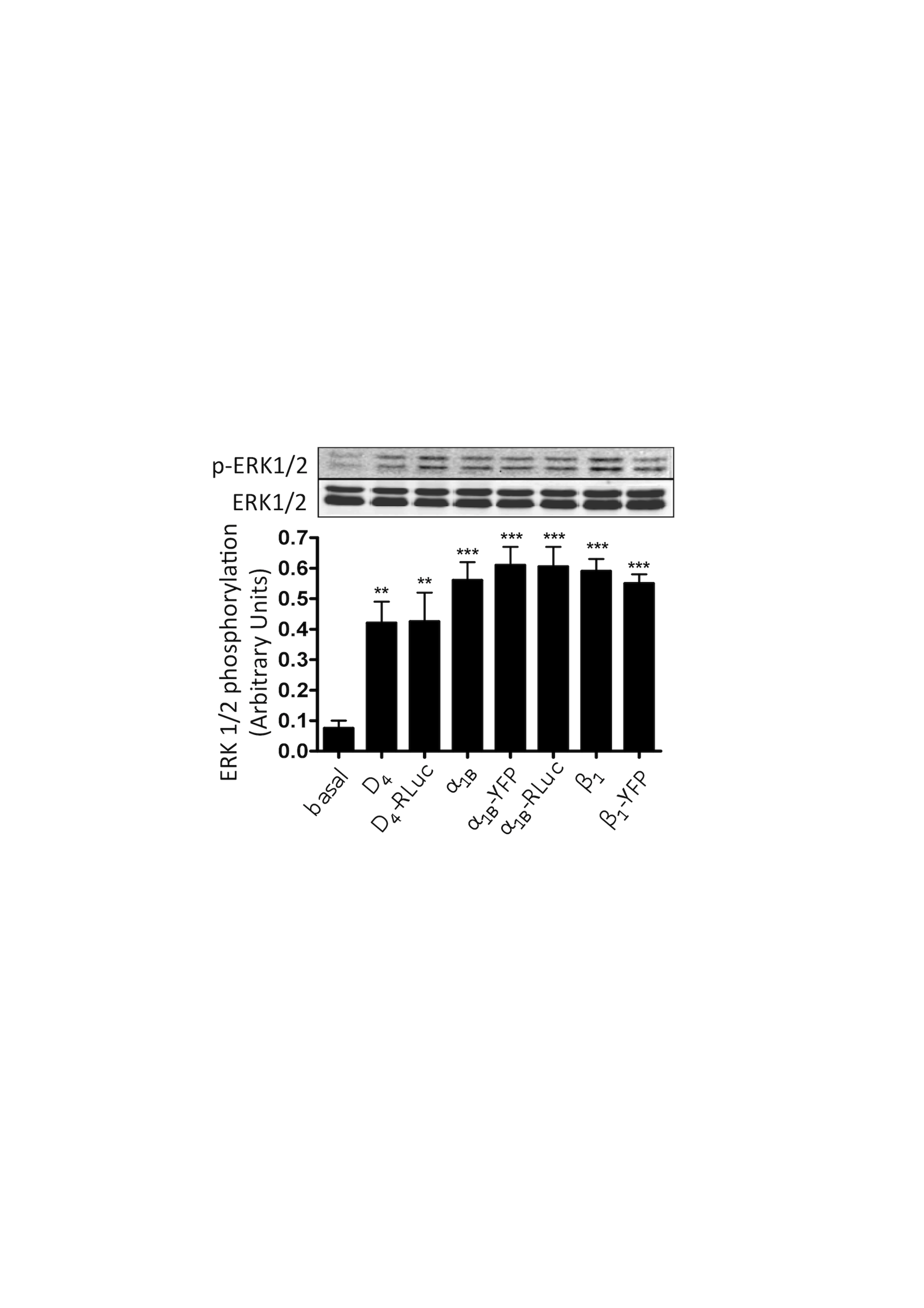

Supplement: Figure S1 — Functionality of the fusion proteins. HEK 293T cells were transfected with 2 µg of plasmid coding for the D4 receptor or with 3 µg of plasmid coding for the adrenergic α1B or β1 receptors or to the corresponding fusion proteins D4-RLuc, α1B-YFP, α1B-RLuc, or β1-YFP. 48 h post-transfection, cells expressing D4 or D4-RLuc receptors were treated with 500 nM RO 10-5824, cells expressing α1B, α1B-YFP or α1B-RLuc receptors were treated with 1 µM phenylephrine, or cells expressing β1 or β1-YFP were treated with 1 µM isoproterenol for 7 min and ERK 1/2 (Thr183-Tyr185) phosphorylation was determined. The immunoreactive bands of three experiments performed in duplicates were quantified and expressed as mean ± S.E.M. of arbitrary units. A representative Western blot is shown at the top. Significant differences with respect to basal levels were calculated by one-way ANOVA followed by a Dunnett's multiple comparison post hoc test (**p<0.01 and ***p<0.001). (TIF) [file pbio.1001347.s001.tif]

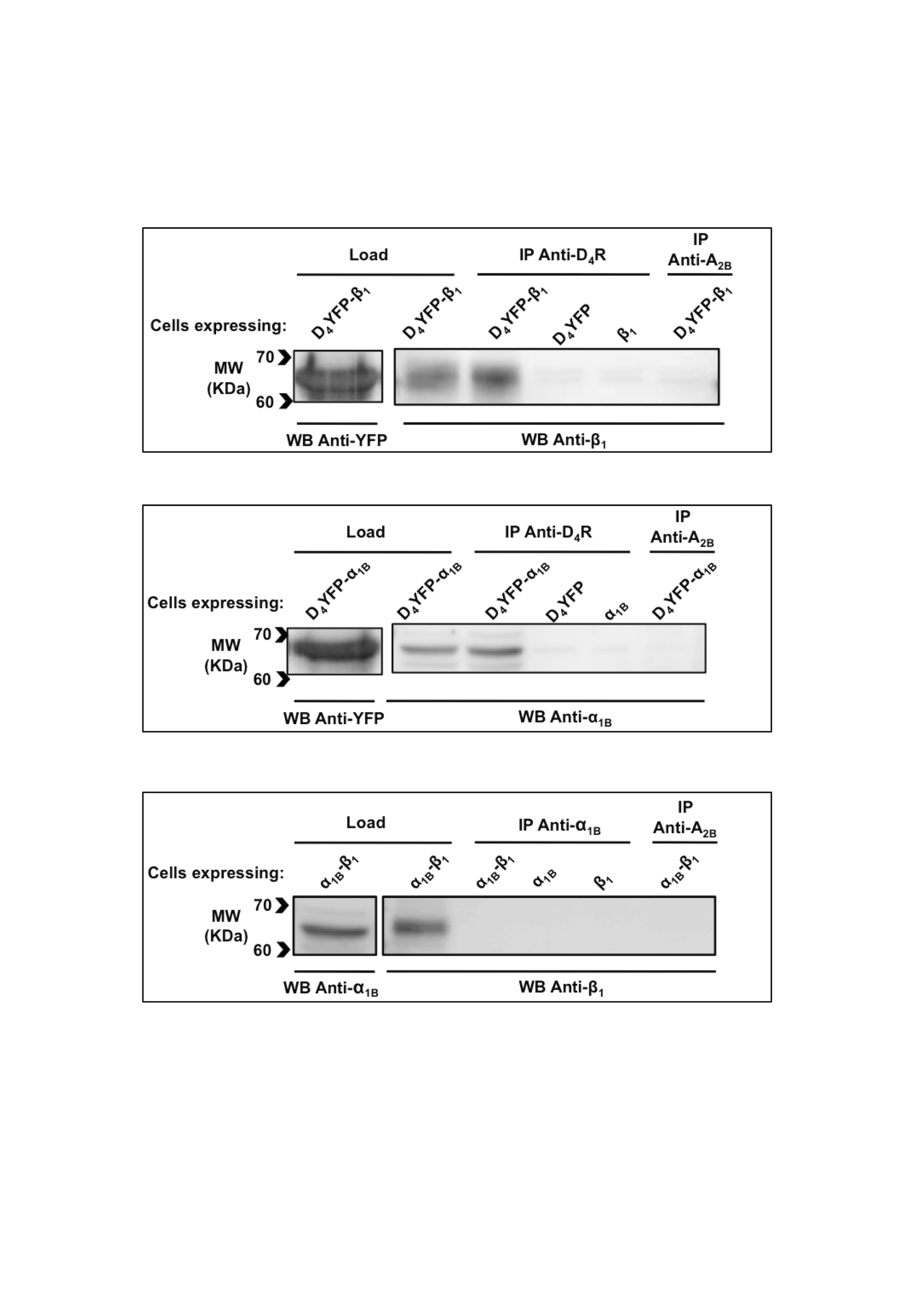

Supplement: Figure S2 — Specificity of the antibodies used for co-immunoprecipitation experiments. Membranes from cells expressing the indicated receptors were solubilized and processed for immunoprecipitation as described under Materials and Methods using goat anti-D4 or rabbit anti-α1 receptor antibodies or goat anti-adenosine A2B or rabbit anti-adenosine A1 receptor antibodies as negative controls. Solubilized membranes (Load) and immunoprecipitates were analyzed by SDS-PAGE and immunoblotted using rabbit anti-YFP, rabbit anti-α1, rabbit anti-β1, or goat anti-β1 receptor antibodies. IP, immunoprecipitation; WB, Western blotting; MW, molecular mass. (TIF) [file pbio.1001347.s002.tif]

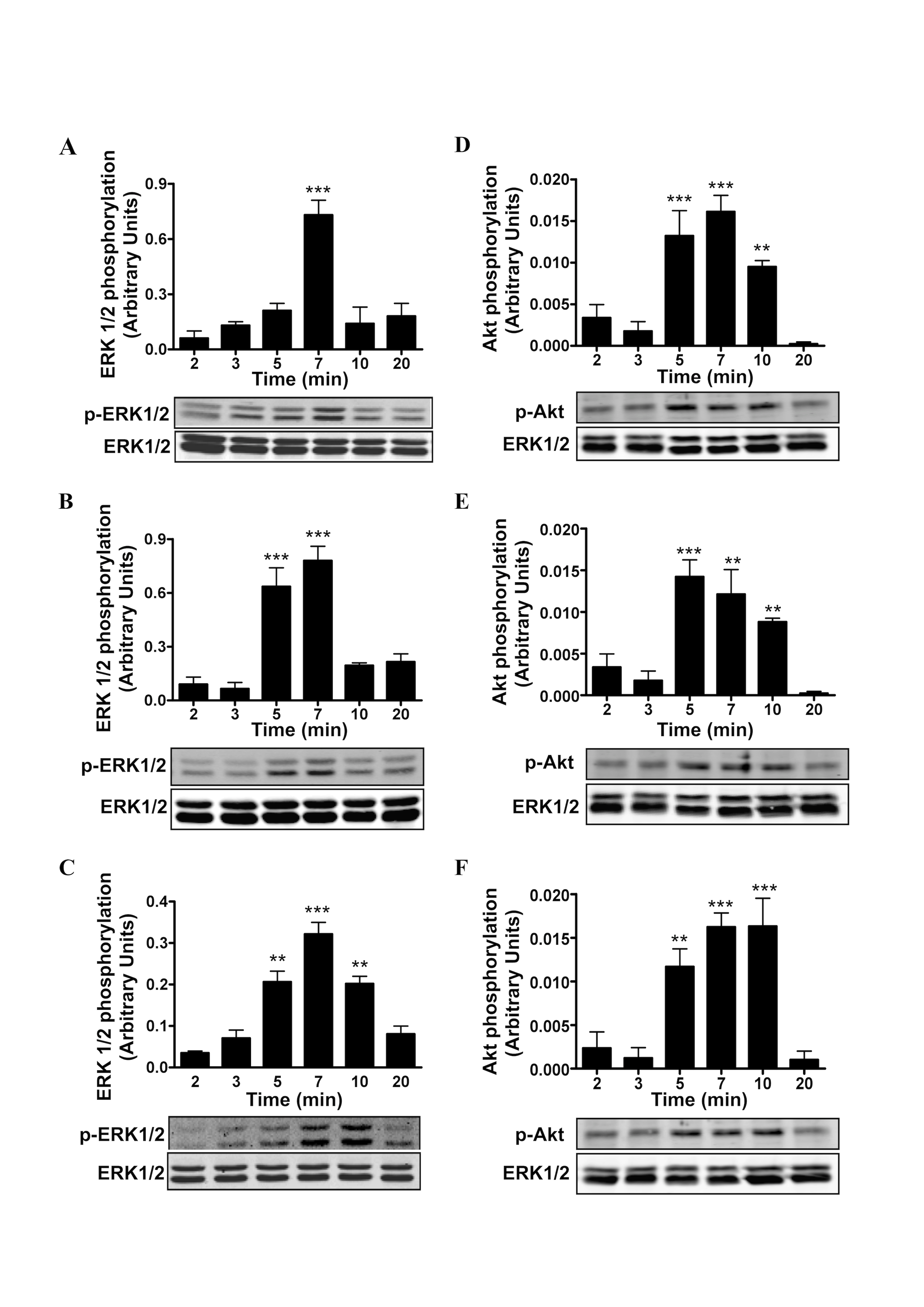

Supplement: Figure S3 — ERK 1/2 and Akt phosphorylation in cells transfected with D4, α1B, or β1 receptors. CHO cells were transfected with 2 µg of plasmid coding for the D4 receptor (A, D), 3 µg of plasmid coding for the α1B receptor (B, E), or 3 µg of plasmid coding for the β1 receptor (C, F). 48 h post-transfection, cells were treated for increasing time with 500 nM RO 10-5824 (A, D), 1 µM phenylephrine (B, E), or 1 µM isoproterenol (C, F). The immunoreactive bands, corresponding to ERK 1/2 (Thr183-Tyr185) (A to C) and Akt (Ser473) (D to F) phosphorylation, of three experiments were quantified and expressed as mean ± S.E.M of arbitrary units. Statistical differences over non-treated cells were determined by one-way ANOVA followed by a Dunnett's multiple comparison post hoc test (*p<0.05, **p<0.01, and ***p<0.001). (TIF) [file pbio.1001347.s003.tif]

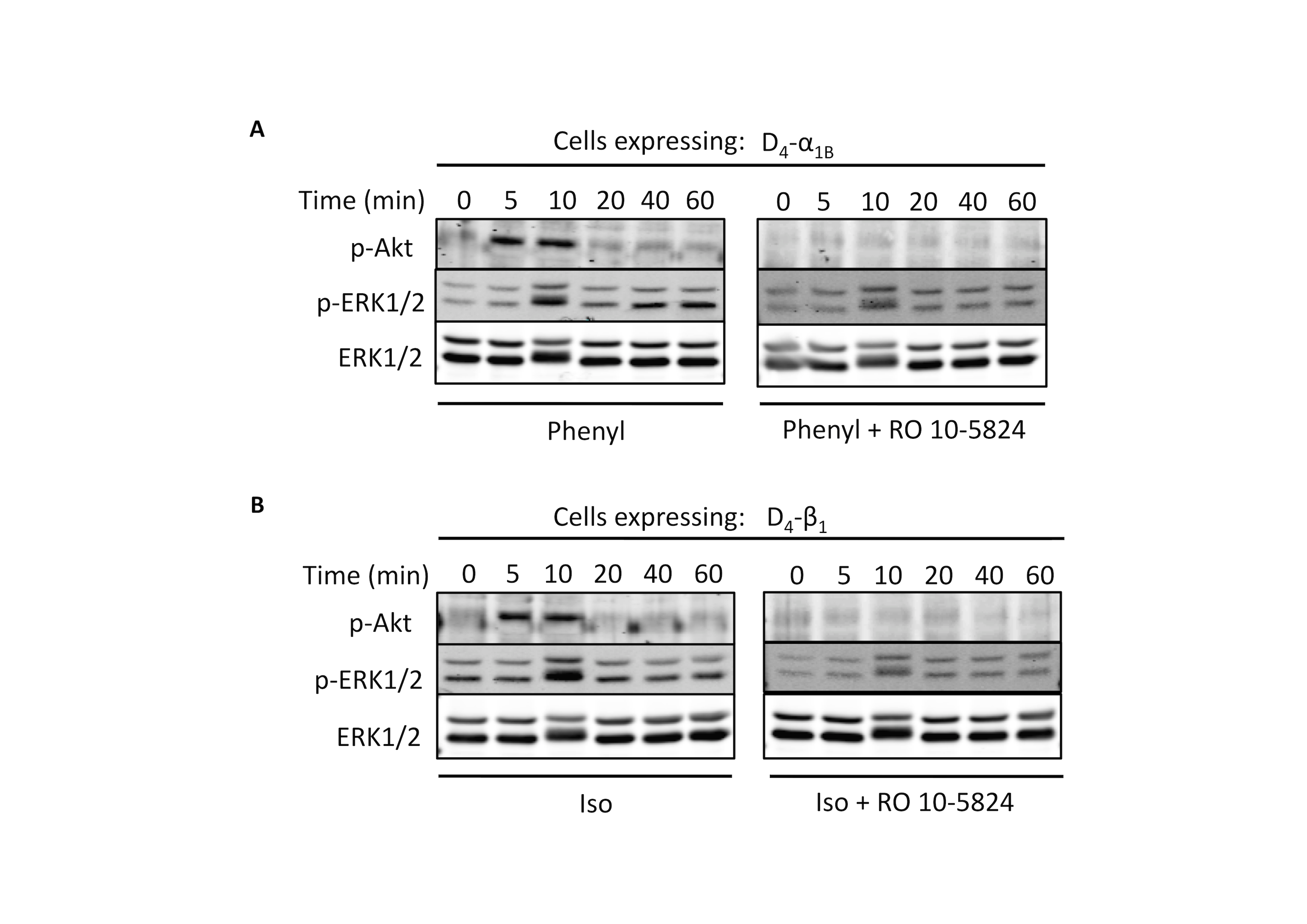

Supplement: Figure S4 — Time-response on ERK 1/2 and Akt phosphorylation by co-activation of α1B-D4 and β1-D4 receptor heteromers in cell cultures. CHO cells were transfected with 2 µg of plasmid coding for the D4 receptor and 3 µg of plasmid coding for the α1B receptor (A) or the β1 receptor (B). 48 h post-transfection, cells were treated with 1 µM phenylephrine (Phenyl, A) or 1 µM isoproterenol (Iso, B) alone or in the presence of 1 µM RO 10-5824 for different times. A representative Western blot is shown. (TIF) [file pbio.1001347.s004.tif]

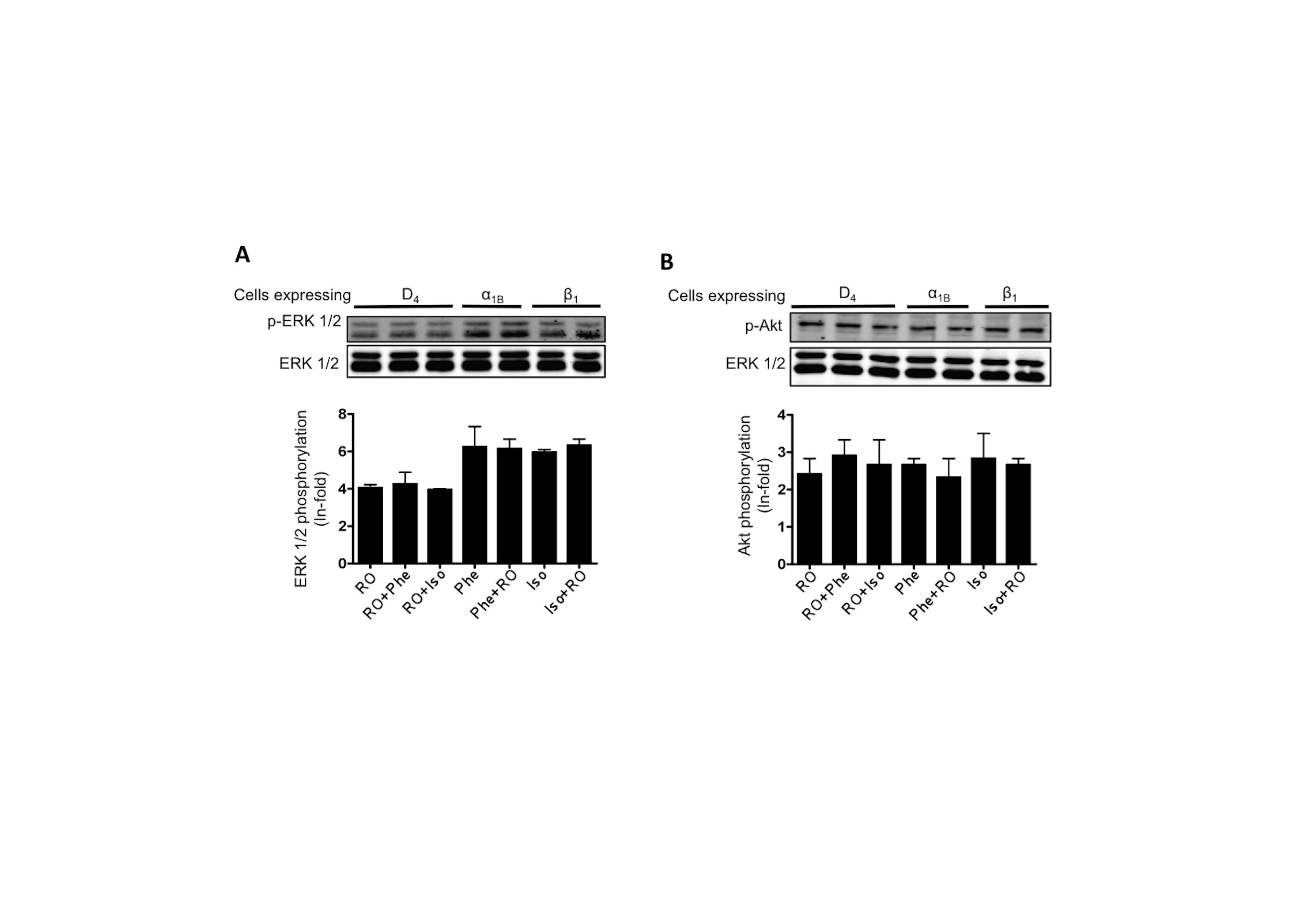

Supplement: Figure S5 — Selectivity of D4, α1B, or β1 receptor agonists. The selectivity of ligands was tested by measuring ERK 1/2 (Thr183-Tyr185) (A) and Akt (Ser473) (B) phosphorylation in cells expressing D4, α1B, or β1 receptors, treated for 7 min with 1 µM RO 10-5824 (RO), phenylephrine (Phe), or isoproterenol (Iso) alone or in combination as indicated. (TIF) [file pbio.1001347.s005.tif]

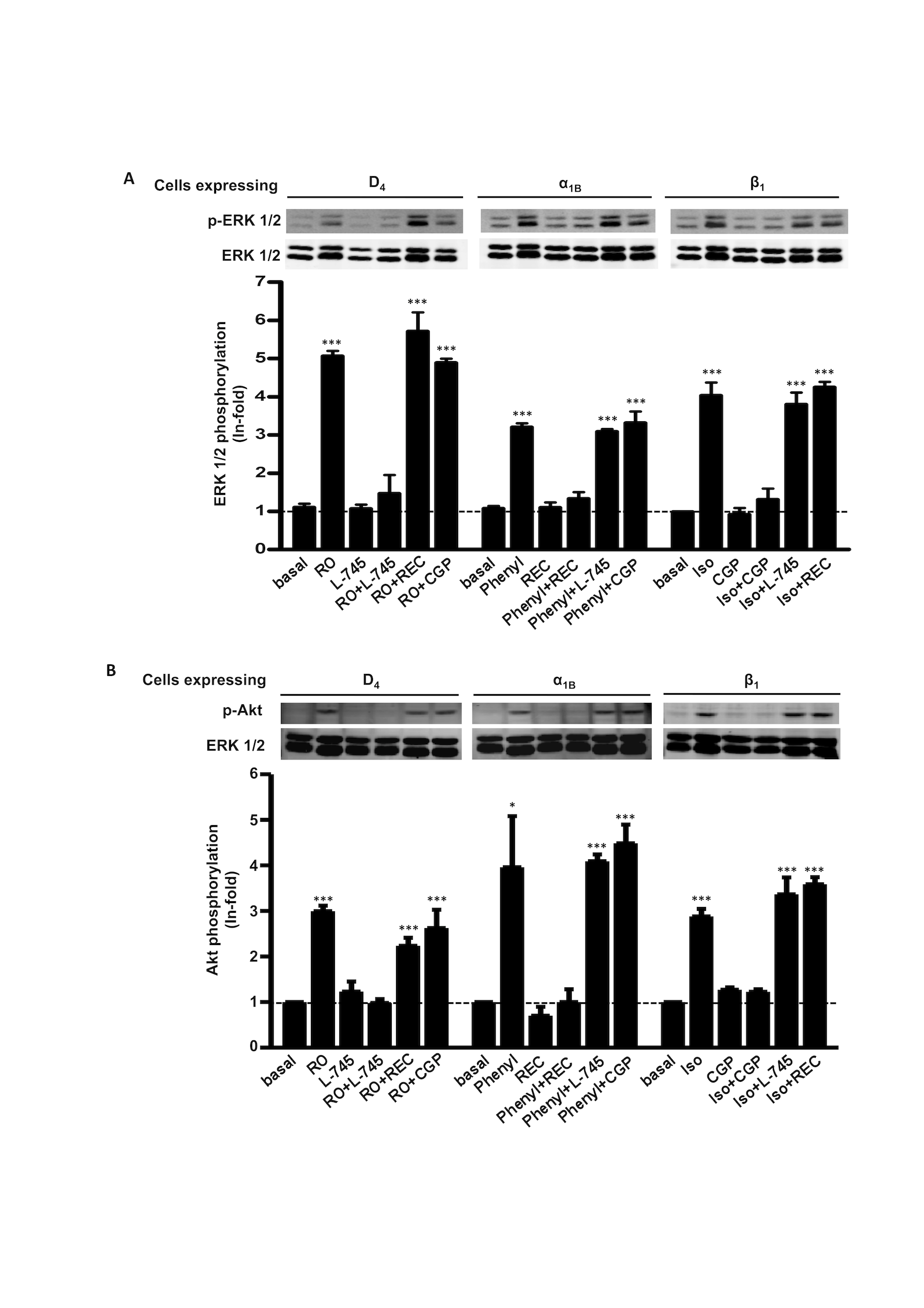

Supplement: Figure S6 — Selectivity of D4, α1B, or β1 receptor antagonists. CHO cells were transfected with 2 µg of plasmid coding for the D4 receptor or with 3 µg of plasmid coding for α1B or β1 receptors. 48 h post-transfection, cells were treated for 7 min with 500 nM RO 10-5824 (RO), 500 nM phenylephrine (Phenyl), 500 nM isoproterenol (Iso), 1 µM L-745,870 (L-745), 1 µM REC 15/2615 (REC), or 1 µM CGP 20712 (CGP) alone or in combination. The immunoreactive bands, corresponding to ERK 1/2 (Thr183-Tyr185) (A) and Akt (Ser473) (B) phosphorylation, of three experiments were quantified and values represent the mean ± S.E.M. of the fold increase over basal levels found in untreated cells (basal). Significant differences over basal levels were determined by one-way ANOVA followed by a Dunnett's multiple comparison post hoc test (*p<0.05, ***p<0.001). A representative Western blot is shown at the top. (TIF) [file pbio.1001347.s006.tif]

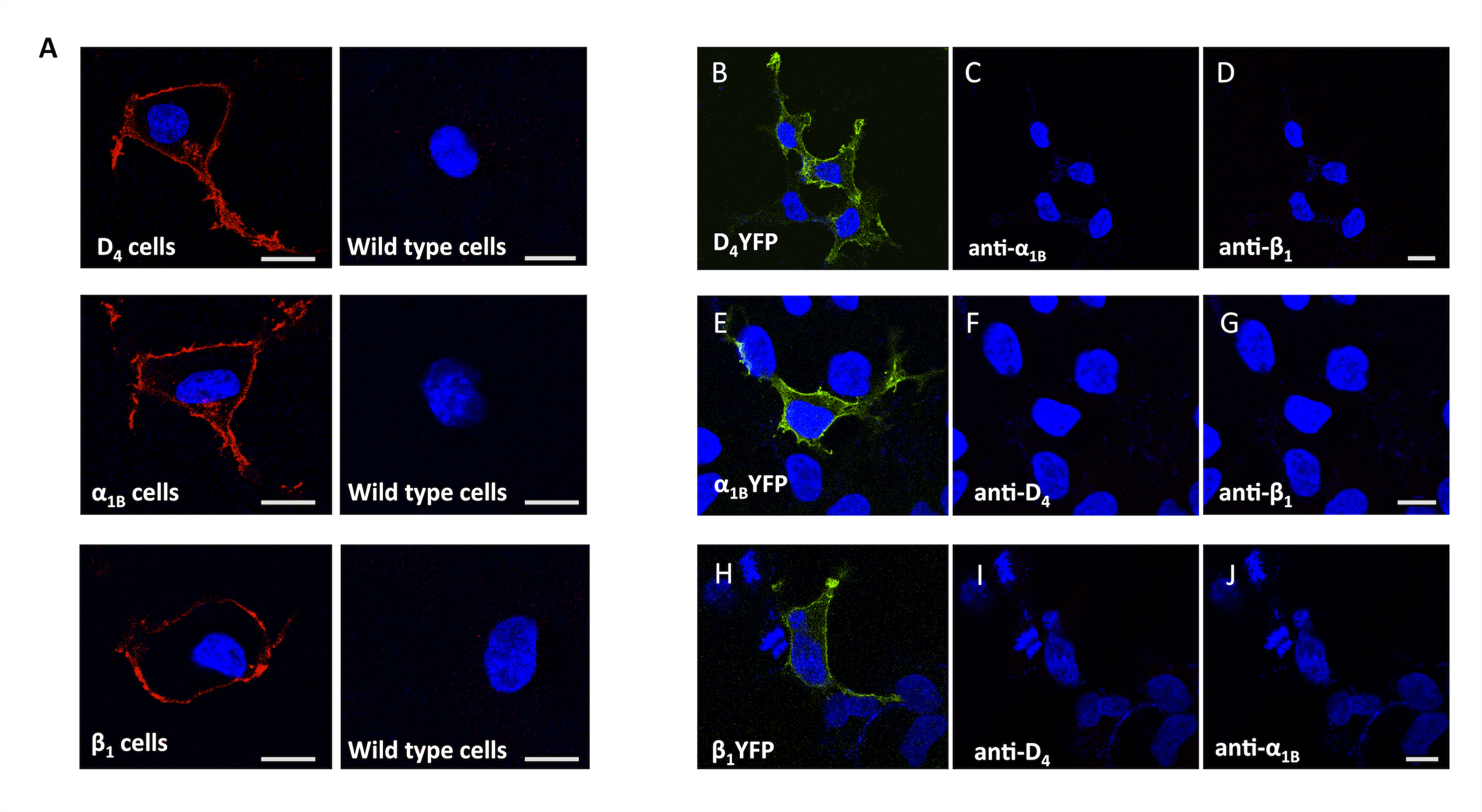

Supplement: Figure S7 — Specificity of the antibodies tested by immunocytochemistry. In (A) non-transfected HEK-293T cells (right panels) and cells transfected with, top to bottom, 1 µg of plasmid coding for D4 receptor, 0.5 µg cDNA coding for α1B receptor, or 0.5 µg cDNA coding for β1 receptor (left panels) were stained using, top to bottom, anti-D4, anti-α1, or anti-β1 antibodies as indicated in Materials and Methods. Scale bar, 5 µm. In (B to J), cells were transfected with 1 µg of plasmid coding for D4-YFP receptor (B to D), 0,5 µg cDNA coding for α1B-YFP receptor (E to G), or 0.5 µg cDNA coding for β1-YFP receptor (H to J). The expression of the receptors was detected by its own YFP fluorescence (B, E, and H) or by using anti-α1 (C and J), anti-β1 (D and G), or anti-D4 (F and I) receptor antibodies. Scale bar, 5 µm. (TIF) [file pbio.1001347.s007.tif]

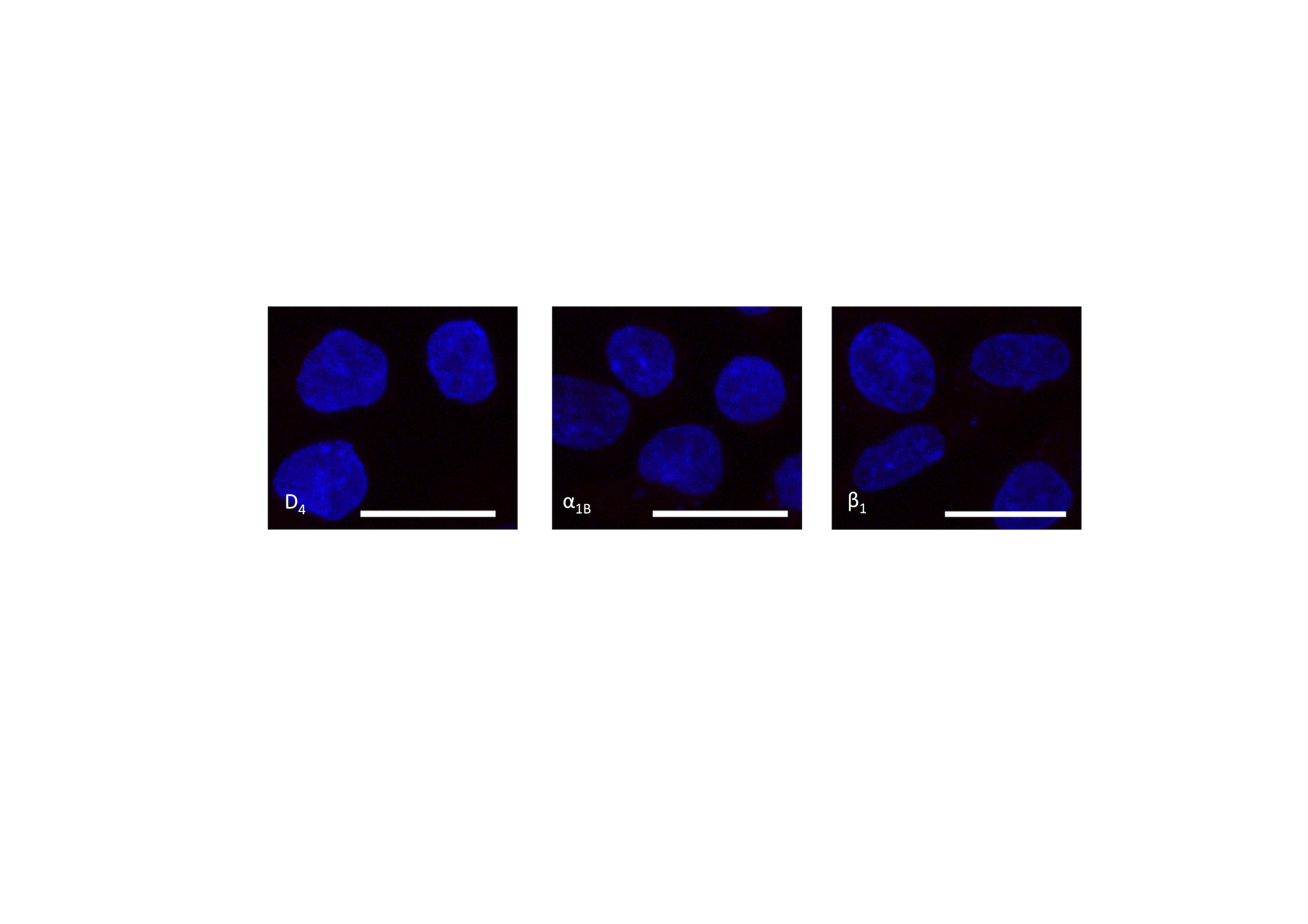

Supplement: Figure S8 — Negative controls for in situ proximity ligation assays. Negative controls for in situ proximity ligation assays (PLA, see Materials and Methods) are shown demonstrating a lack of punctate red fluorescence staining in pinealocytes in the absence of primary antibodies, left to right, anti-D4, anti-α1, or anti-β1 antibodies. Scale bar, 20 µm. (TIF) [file pbio.1001347.s008.tif]

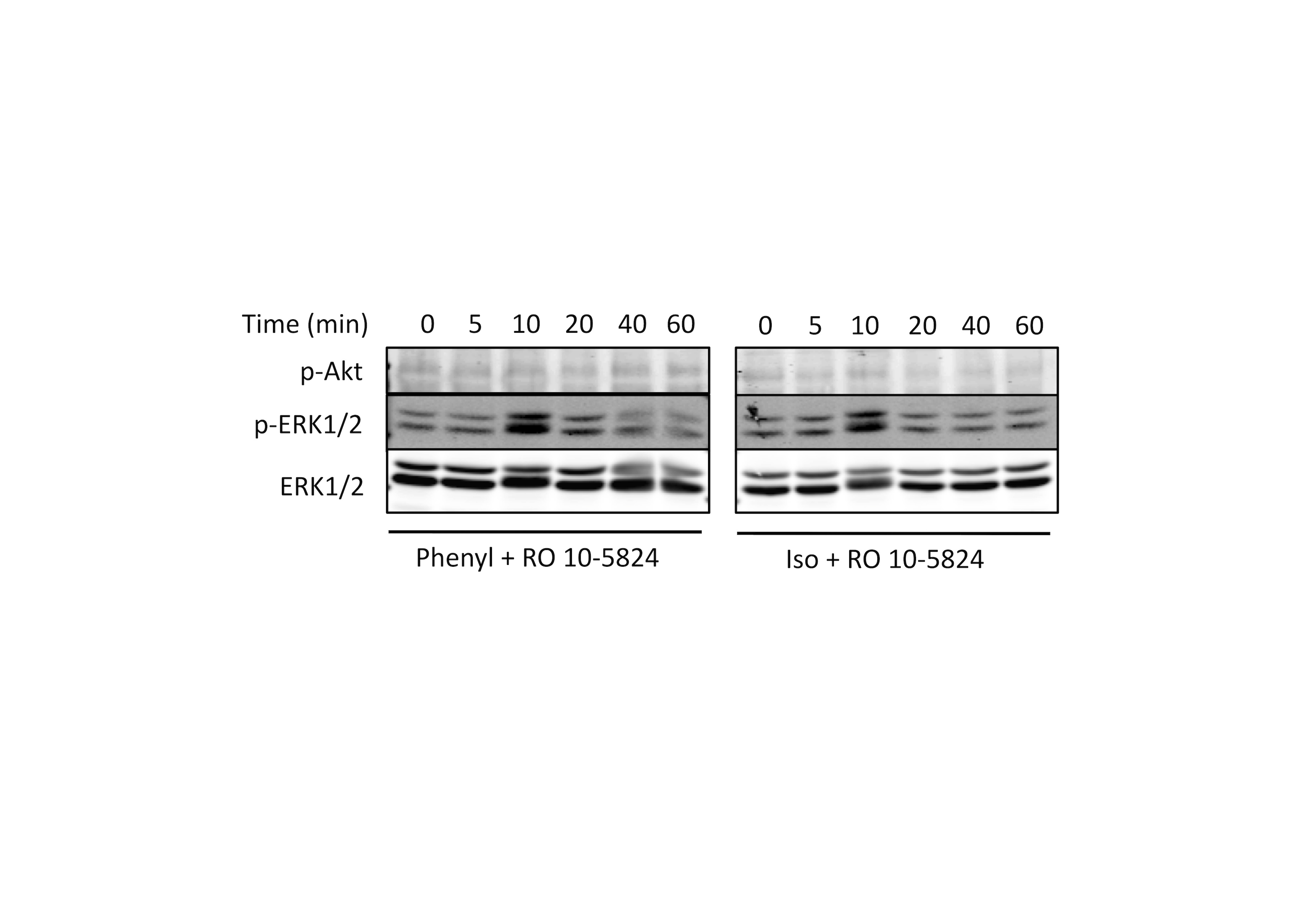

Supplement: Figure S9 — Time-response on ERK 1/2 and Akt phosphorylation by co-activation of α1B-D4 and β1-D4 receptor heteromers in pineal gland. Pineal glands extracted at 9:00 h were treated with 1 µM phenylephrine (Phenyl) or 1 µM isoproterenol (Iso) in the presence of 1 µM RO 10-5824 for the times indicated. A representative Western blot is shown. (TIF) [file pbio.1001347.s009.tif]
