# Supplementary material for: Circadian-Related Heteromerization of Adrenergic and Dopamine D4 Receptors Modulates Melatonin Synthesis and Release in the Pineal Gland
Source: PLoS Biol. 2012 Jun 19;10(6):e1001347. doi: 10.1371/journal.pbio.1001347 (PMC3378626; doi:10.1371/journal.pbio.1001347)
Supplement: Text S1 — Additional details on materials and methods used throughout the article. (DOC) [file pbio.1001347.s010.doc]

**Text S1**

**Fusion proteins and expression vectors.** The cDNA for the human dopamine D_4_ receptor expressed in the *pcDNA3.1* vector (Invitrogen, Paisley, Scotland, UK) was amplified without its stop codon using sense and antisense primers harboring unique XhoI and EcoRI sites to be cloned it in the mammalian humanized pRluc-N1 vectors (Perkin-Elmer, Waltham, MA, USA). The cDNA for the human adrenergic α_1B_ receptor gene, cloned in pOmicsLink ORF Expression Clone (GeneCopoeia, Maryland, USA) was amplified without its stop codon using sense and antisense primers harbouring unique KpnI and ApaI restriction sites, to be subcloned into KpnI/ApaI sites of the *pcDNA3.1* vector, the pRluc-N1 vector or the EYFP-N3 vector (enhanced yellow variant of YFP; Clontech, Heidelberg, Germany). Finally, the cDNA for the human adrenergic β_1_ receptor gene, (kindly provided by Dr. S. Dorsch, University of Wuerzburg, Germany) and D_1_ receptor cloned in *pcDNA3.1* vector were amplified without their stop codon using sense and antisense primers harboring unique BamHI and HindIII sites or EcoRI and KpnI, respectively to be cloned in the EYFP-N3 vector. The resulting plasmids express the receptors fused to either Rluc or YFP on the C-terminal end of the receptor (D_4_-RLuc, α_1B_-RLuc, α_1B_-YFP, β_1_-YFP, and D_1_-YFP, respectively). All constructs were verified by nucleotide sequencing and the fusion proteins were functional and expressed at the membrane level (see Results).

**Cell culture and transient transfection.** CHO cell were maintained in α-MEM medium without nucleosides (Invitrogen), containing 10% fetal calf serum, 50 μg/ml penicillin, 50 μg/ml streptomycin and 2 mM L-glutamine. Human embryonic kidney (HEK)-293T cells were grown in Dulbecco’s modified Eagle’s medium (DMEM) supplemented with 2 mM L-glutamine, 100 U/ml penicillin/streptomycin, and 5% (v/v) heat inactivated Foetal Bovine Serum (FBS) (all from Invitrogen). Cells were maintained at 37ºC in an atmosphere of 5% CO_2_, and were passaged every 3 or 4 days when they were 80-90% confluent. HEK-293T or CHO cells growing in 6-well dishes or in 25 cm^2^ flasks were transiently transfected with the corresponding fusion protein cDNA by the polyethylenimine (PEI) (PolyEthylenImine, Sigma, Steinheim, Germany) method. Cells were incubated (4 h) with the corresponding cDNA together with ramified PEI (5 ml of 10 mM PEI for each mg cDNA) and 150 mM NaCl in a serum-starved medium. After 4 h, the medium was changed to a fresh complete culture medium. Forty-eight hours after transfection, cells were washed twice in quick succession in Hanks' balanced salt solution HBSS (137 mM NaCl, 5 mM KCl, 0.34 mM Na_2_HPO_4_·12H_2_O, 0.44 mM KH_2_PO_4_, 1.26 mM CaCl_2_·2H_2_O, 0.4 mM MgSO_4_·7H_2_O, 0.5 mM MgCl_2_, 10 mM HEPES, pH 7.4) supplemented with 0.1% glucose (w/v), detached, and resuspended in the same buffer. To control the cell number, sample protein concentration was determined using the Bradford assay kit (Bio-Rad, Munich, Germany) using bovine serum albumin dilutions as standards.

**Immunostaining.** For immunocytochemistry, HEK-293T cells were grown on glass coverslips and transiently transfected as indicated in the Figure legends. After 48h of transfection cells were fixed in 4% paraformaldehyde for 15 min and washed with phosphate-buffered saline (PBS) containing 20 mM glycine to quench the aldehyde groups. After permeabilization with PBS containing 0.05% Triton X-100 for 15 min, cells were incubated 1 h at room temperature with PBS containing 1% bovine serum albumin and were labeled with the primary goat polyclonal anti-D_4_ receptor antibody (1/500, Santa Cruz Biotechnology), rabbit anti-α_1_ receptor antibody (1:100, Abcam, Cambridge, UK) or rabbit anti-β_1_ receptor antibody (1:100, Santa Cruz Biotechnology) for 1 h, washed and stained with the secondary antibody Cy3 labeled anti-goat (1/200, Jackson ImmunoResearch, Baltimore, PA) or Cy3 labeled anti-rabbit (1/200, Jackson ImmunoResearch, Baltimore, PA). The D_4_-YFP, α_1B_-YFP and β_1_-YFP constructs were detected by monitoring fluorescence emission at 530 nm. Samples were rinsed and observed using an Olympus FV1000 confocal microscope.

**BRET assay.** HEK-293T cells were co-transfected with a constant amount of cDNA encoding for the receptor fused to Rluc and with increasing amounts of cDNA encoding the receptor fused to YFP and used after 48 h of transfection. With aliquots of transfected cells (20 µg of total protein), three different determinations were performed in parallel: i) To quantify fluorescence protein expression, cells were distributed in 96-well microplates (black plates with a transparent bottom, Porvair, King´s Lynn, UK),) and fluorescence was read in a Fluostar Optima Fluorimeter (BMG Labtechnologies, Offenburg, Germany) equipped with a high-energy xenon flash lamp, using a 10 nm bandwidth excitation filter at 400 nm reading. Receptor-fluorescence expression was determined as fluorescence of the sample minus the fluorescence of cells expressing receptor-Rluc alone. ii) For BRET measurements, the equivalent of 20 µg of cell suspension were distributed in 96-well microplates (Corning 3600, white plates with white bottom, Sigma) and 5 µM coelenterazine H (Molecular Probes, Eugene, OR) was added. After 1 minute of adding coelenterazine H, the readings were collected using a Mithras LB 940 that allows the integration of the signals detected by a filter at 485 nm and a filter at 530 nm. iii) To quantify receptor-Rluc expression luminescence readings were also performed after 10 minutes of adding 5 µM coelenterazine H. Both fluorescence and luminescence for each sample were measured before every experiment to confirm similar donor expressions (approximately 100,000 bioluminescence units) while monitoring the increase in acceptor expression (2000 to 40,000 fluorescence units). The relative amounts of BRET acceptor are expressed as the ratio between the net fluorescence of the acceptor and the luciferase activity of the donor being the net fluorescence the fluorescence of the acceptor minus the fluorescence detected in cells only expressing the donor. The BRET ratio is defined as [(emission at 510-590)/(emission at 440-500)] - Cf, where Cf corresponds to (emission at 510-590)/(emission at 440-500) for the D_4_-RLuc construct expressed alone in the same experimental conditions. BRET was expressed as mili BRET Units (mBU) and is the BRET ratio x 1000. Curves were fitted by using a non-linear regression equation, assuming a single phase with GraphPad Prism software (San Diego, CA, USA).

**Coimmunoprecipitation.** Transfected cells or pineal glands were disrupted with a Polytron homogenizer in 50 mM Tris-HCl buffer, pH 7.4, containing a protease inhibitor mixture (1/ 1000, Sigma). The cellular debris was removed by centrifugation at 13,000 g for 5 min at 4 °C, and membranes were obtained by centrifugation at 105,000 g for 1 h at 4 °C. Membranes were washed two more times at the same conditions and were solubilized by homogenization in ice-cold immunoprecipitation buffer (phosphate-buffered saline, pH 7.4, containing 1% (v/v) Nonidet P-40) and incubated for 30 min on ice before centrifugation at 105,000 g for 1 h at 4 °C. The supernatant (1 mg/ml of protein) was processed for immunoprecipitation as described in the immunoprecipitation protocol using a Dynabeads Protein G kit (Invitrogen) using goat anti-D_4_ receptor antibody (1:1000, Santa Cruz Biotechnology), rabbit anti-α_1_ receptor antibody (1:1000, Abcam), rabbit anti-β_1_ receptor antibody (1:1000, Santa Cruz Biotechnology) or goat anti-β_1_ receptor antibody (1:1000, Abcam). As negative control goat anti-adenosine A_2B_ receptor antibody (1:1000, Santa Cruz Biotechnology) or rabbit anti-adenosine A_1_ receptor antibody (1:1000, Affinity BioReagents, Rockford USA) was used. Protein was quantified by the bicinchoninic acid method (Pierce) using bovine serum albumin dilutions as standard. Immunoprecipitates were separated on a denaturing 10% SDS-polyacrylamide gel and transferred onto PVDF membranes. Membranes were blocked for 90 min in 5% milk and PBS-Tween 20 (0.05% V/V). The following primary antibodies were incubated overnight at 4°C in 5% milk and PBS-Tween 20 (0.05% V/V): rabbit anti-YFP antibody (1:800, Santa Cruz Biotechnology), rabbit anti-α_1_ receptor antibody (1:800, Abcam), rabbit anti-β_1_ receptor antibody (1:500, Santa Cruz Biotechnology) or goat anti-β_1_ receptor antibody (1:800, Abcam). The following day the membranes were washed 3 times for 10 minutes in PBS Tween-20 (0.05% V/V) and the secondary antibodies: mouse anti-rabbit-peroxidase (1:20,000, Sigma) or donkey anti-goat-peroxidase (1:20,000, Jackson ImmunoResearch Laboratories, West Grove, PA) incubated for 1 hr at room temperature in 5% milk and PBS-Tween 20 (0.05% V/V). Following an additional three washes for 10 min each with PBS Tween-20 (0.05% V/V) and a final wash with PBS, bands were detected with the addition of SuperSignal West Pico Chemiluminescent Substrate (Pierce) and visualized with a LAS-3000 (Fujifilm). Analysis of detected bands was performed by Image Gauge software (version 4.0) and Multi Gauge software (version 3.0).

**MAPK and Akt/PKB determination**. Transfected CHO cells or pineal glands were cultured in serum-free medium for 16h before the addition of the indicated concentration of ligands for the indicated time. Both, cells and pineal glands were rinsed with ice-cold PBS and lysed by the addition of 300μl of ice-cold lysis buffer (50mM Tris-HCl pH 7.4, 50mM NaF, 150 mM NaCl, 45 mM β-glycerophosphate, 1% Triton X-100, 20 mM phenylarsine oxide, 0.4 mM NaVO_4_ and protease inhibitor cocktail) and by shaking (cells) or sonicating (pineal glands, Branson Digital Sonifier S-250 from Branson Ultrasonic Corporation, Dambury, USA with an amplitude of 10% for 10 seconds). Cellular debris was removed by centrifugation at 13,000 g for 5 min at 4ºC and protein was quantified by the bicinchoninic acid method using bovine serum albumin as a standard. To determine the level of MAPK (ERK1/2) and Akt/PKB phosphorylation, equivalent amounts of protein (10μg) were separated by electrophoresis on a denaturing 10% SDS-polyacrylamide gel and transferred onto PVDF-FL membranes. Odyssey blocking buffer (LICOR Biosciences, Lincoln, Nebraska, USA) was then added and membranes were blocked for 90 min. Membranes were then probed for 2-3 h with a mixture of a mouse anti-phospho-Thr^183^-Tyr^185^-ERK 1/2 antibody (1:2500, Sigma, Steinheim, Germany), a rabbit anti-phospho-Ser^473^-Akt antibody (1/2500, SAB Signalway Antibody, Pearland, USA) and a rabbit anti-ERK 1/2 antibody (1:40000, Sigma, Steinheim, Germany) to control differences in loading. Bands were visualized by the addition of a mixture of IRDye 800 (anti-mouse) antibody (1:10000, Sigma) and/or IRDye 680 (anti-rabbit) antibody (1:10000, Sigma) for 1 h and scanned by the Odyssey infrared scanner. Bands densities were quantified using the scanner software and exported to Excel (Microsoft, Redmond, WA, USA). The level of phosphorylated ERK 1/2 isoforms or phosphorylated Akt in the same membrane were normalized for differences in loading using the total ERK protein band intensities.

**Radioligand Binding Experiments.** Competition experiments were performed. Membrane suspensions (2 mg of protein/ml) from CHO cells expressing D_4_ and α_1B_ or β_1_ receptors, were incubated for 2 h at 25°C in 50 mM Tris-HCl buffer, pH 7.4, containing 10 mM MgCl_2_ with the indicated concentration of the α_1B_ receptor antagonist [^3^H]prazosin or β_1_ receptor antagonist [^3^H]CGP-12177 (PerkinElmer Life and Analytical Sciences) and increasing concentrations of phenylephrine or isoproterenol respectively, in the absence or in the presence of the indicated concentration of the D_4_ receptor agonist RO 10-5824 (Tocris, Aronmouth, UK). Free and membrane-bound ligand were separated by rapid filtration of 500 μl aliquots in a cell harvester (Brandel, Gaithers- burg, MD) through Whatman GF/C filters embedded in 0.3% polyethylenimine that were subsequently washed for 5 s with 5 ml of ice-cold Tris-HCl buffer. The filters were incubated with 10 ml of Ecoscint H scintillation cocktail (National Diagnostics, Atlanta, GA) overnight at room temperature, and radioactivity counts were determined by use of a Tri-Carb 1600 scintillation counter (PerkinElmer Life and Analytical Sciences) with an efficiency of 62%.

Competition curves were analyzed by non-linear regression, using the commercial Grafit software (Erithacus Software), by fitting the binding data to the two-state dimer receptor model [1,2]. To calculate the macroscopic equilibrium dissociation constants the equations deduced by Casadó et al. were used [3]. Goodness of fit was tested according to reduced chi-squared value given by the non-linear regression program.

**Pinealocyte culture, signaling and immunocytochemistry**. Pinealocytes were prepared from rat pineal glands as previously described by Silveira Cruz-Machado et al. [4]. Briefly, pinealocytes were obtained by trypsinization (0.25%, 37°C, 15 min) followed by mechanical dispersion in the presence of fetal bovine serum. Cells were pelleted and resuspended in BGJb medium supplemented with 10% v/v fetal bovine serum (heat- inactivated), 100 U/mL penicillin/streptomycin (pH 7.4). The total number of cells and fractional survival was estimated by Trypan blue exclusion. Cells (200.000 x well) were plated on polylysine coated 6-well chamber plate and maintained at 37°C, 5% CO_2_ for 48 h prior to use. For signalling experiments, pinealocytes were treated with specific agonist for 10 min, fixed in 4% paraformaldehyde for 15 min and washed with PBS containing 20 mM glycine. After permeabilization with PBS containing 0.05% Triton X-100 for 15 min, pinealocytes were treated 1 h at room temperature with PBS containing 1% bovine serum albumin and were labeled with the mouse monoclonal anti-S-arrestin 2 (1/100, Thermo Scientific, Rockford, USA) and the rabbit polyclonal anti-phospho-Thr^202^/Tyr^204^ ERK1/2 (1/300, Cell Signaling Technology, Danvers, MA) for 1 h, washed and stained with the secondary chicken anti-rabbit (1/200, Alexa Fluor 594, Invitrogen) and goat anti-mouse (1/200, Alexa Fluor 488, Invitrogen). For D_4_, α_1B_ or β_1_ receptor staining pinealocytes were labeled with the goat anti D_4_ receptor antibody (1:100, Santa Cruz Biotechnology, Heidelberg, Germany), rabbit anti-α_1_ receptor antibody (1:100, Abcam, Cambridge, UK) or rabbit anti-β_1_ receptor antibody (1:100, Santa Cruz Biotechnology) and mouse monoclonal anti-S-arrestin 2 (1/100, Thermo Scientific) and the secondary antibody Cy3 labeled anti-goat (1/200, Jackson ImmunoResearch, Baltimore, PA) or Cy3 labeled anti-rabbit (1/200, Jackson ImmunoResearch) and Cy3 labeled anti-mouse (1/200, Jackson ImmunoResearch . Coverslips were rinsed with PBS three times at room temperature and fluorescence was observed in a Leica SP2 confocal microscope equipped with an Argon UV lamp with excitation at 351nm or 364nm, an Argon laser at 488nm and a DPSS laser at 561nm. A 63X oil objective was used.

**In Situ Proximity Ligation Assay (PLA)**. The primary cultures of pinealocytes were fixed and permeabilized as described above. The receptor-receptor molecular interaction was detected using the Duolink II in situ PLA detection Kit (OLink; Bioscience, Uppsala, Sweden). After 1 h incubation at 37°C with the blocking solution in a pre-heated humidity chamber, pinealocytes were incubated overnight with the primary antibodies: goat anti-D_4_ antibody (1:100, Santa Cruz Biotechnology, Heidelberg, Germany) and rabbit anti-α_1_ antibody (1:100, Abcam, Cambridge, UK) to detect α_1B_-D_4_ receptor heteromers, goat anti-D_4_ antibody and rabbit anti β_1_ antibody (1:100, Santa Cruz Biotechnology) to detect β_1_-D_4_ receptor heteromers or rabbit anti-α_1_ antibody and goat anti-β_1_ antibody (1:100, Abcam) to detect α_1B_-β_1_ receptor heteromers, in the antibody diluent medium. The pinealocytes were washed with buffer A ( 10 mM Tris, 150mM NaCl, and 0.05% (v/v )Tween-20,) at room temperature and incubated for 2 h in a pre-heated humidity chamber at 37°C with PLA probes detecting rabbit or goat antibodies (Duolink II PLA probe anti-Rabbit plus and Duolink II PLA probe anti-Goat minus diluted in the antibody diluent to a concentration of 1:5). After washing with wash buffer A at room temperature, pinealocytes were incubated in a pre-heated humidity chamber for 30 min at 37°C, with the ligation solution (Duolink II Ligation stock 1:5 and Duolink II Ligase 1:40). Detection of the amplified probe was done with the Duolink II Detection Reagents Red Kit. After exhaustively washing at room temperature with wash buffer B (200 mM Tris and 100 mM NaCl), the pinealocytes were mounted using the mounting medium with DAPI. The samples were observed in a Leica SP2 confocal microscope. As negative controls for the technique, the same procedure was done but omitting the primary antibodies. As negative control for heteromerization, heteromers between α_1B_ and β_1_ receptors were tested.

**Serotonin synthesis and release determination.** After 36 h of culture in BGJb medium (Invitrogen, Carlsbad, CA), the pineal glands were incubated in HBSS medium supplemented with 0,1% glucose, 100 U/ml penicillin/streptomycin and 1mg/ml bovine serum albumin for 12 h with specific agonist and/or antagonist and radioactive [^14^C]-Tryptophan (10 μM). After incubation, medium and pineal glands were collected separately into eppendorf tubes with 35 μl of trichloroacetic acid (TCA 1%) and were kept at 4ºC. Pineal glands were homogenized in a Dynatech/Sonic Dismembrator (Dynatech Labs, Chantilly, VA) for 15 seconds. An aliquot was reserved for protein quantification by the Lowry method and cellular debris were removed by centrifugation at 10,000 g for 10 min at 4ºC. [^14^C]-Serotonin present in the supernatant was separated from [^14^C]-Tryptophan by HPLC coupled to detection by fluorescence (excitation: 252nm; emission:382). The chromatography system consisted of a reverse-phase C18 column (2.5μm particle Fortis C18, 100 x 4.6, Sugelabor, Spain) and an ion-pair mobile phase, made up of 500mM sodium acetate, 500mM citric acid, 1mM EDTA, 5 mM octanesulfonic acid plus 20% methanol (v/v) , pH 3.8. The flow rate was 1ml/min. Serotonin fractions were recovered in scintillation vials, mixed with Optiphase HiSafe III cocktail, and [^14^C]-serotonin was quantified in a liquid scintillation counter.

**References**

1. Franco R, Casadó V, Mallol J, Ferré S, Fuxe K, et al. (2005) Dimer-based model for heptaspanning membrane receptors. Trends Biochem Sci 30: 360–366. doi:10.1016/j.tibs.2005.05.010.

2. Franco R, Casadó V, Mallol J, Ferrada C, Ferré S, et al. (2006) The two-state dimer receptor model: a general model for receptor dimers. Mol Pharmacol 69: 1905–1912. doi:10.1124/mol.105.020685.

3. Casadó V, Ferrada C, Bonaventura J, Gracia E, Mallol J, et al. (2009) Useful pharmacological parameters for G-protein-coupled receptor homodimers obtained from competition experiments. Agonist-antagonist binding modulation. Biochem Pharmacol 78: 1456–1463. doi:10.1016/j.bcp.2009.07.012.

4. Da Silveira Cruz‐Machado S, Carvalho‐Sousa CE, Tamura EK, Pinato L, Cecon E, et al. (2010) TLR4 and CD14 receptors expressed in rat pineal gland trigger NFKB pathway. Journal of Pineal Research 49: 183–192. doi:10.1111/j.1600-079X.2010.00785.x.
